# The long journey of *Orthotrichum shevockii* (Orthotrichaceae, Bryopsida): From California to Macaronesia

**DOI:** 10.1371/journal.pone.0211017

**Published:** 2019-02-13

**Authors:** Beatriz Vigalondo, Jairo Patiño, Isabel Draper, Vicente Mazimpaka, James R. Shevock, Ana Losada-Lima, Juana M. González-Mancebo, Ricardo Garilleti, Francisco Lara

**Affiliations:** 1 Departamento de Biología (Botánica), Facultad de Ciencias, Universidad Autónoma de Madrid, Madrid, Spain; 2 Departamento de Botánica, Ecología y Fisiología Vegetal, Universidad de La Laguna, La Laguna, Santa Cruz de Tenerife, Spain; 3 Island Ecology and Evolution Research Group, Instituto de Productos Naturales y Agrobiología (IPNA-CSIC), La Laguna, Tenerife, Spain; 4 Department of Environmental Science, Policy and Management, University of California, Berkeley, CA, United States of America; 5 Department of Botany, California Academy of Sciences, San Francisco, CA, United States of America; 6 Departamento de Botánica y Geología, Facultad de Farmacia, Universidad de Valencia, Valencia, Spain; Austrian Federal Research Centre for Forests BFW, AUSTRIA

## Abstract

Biogeography, systematics and taxonomy are complementary scientific disciplines. To understand a species’ origin, migration routes, distribution and evolutionary history, it is first necessary to establish its taxonomic boundaries. Here, we use an integrative approach that takes advantage of complementary disciplines to resolve an intriguing scientific question. Populations of an unknown moss found in the Canary Islands (Tenerife Island) resembled two different Californian endemic species: *Orthotrichum shevockii* and *O*. *kellmanii*. To determine whether this moss belongs to either of these species and, if so, to explain its presence on this distant oceanic island, we combined the evaluation of morphological qualitative characters, statistical morphometric analyses of quantitative traits, and molecular phylogenetic inferences. Our results suggest that the two Californian mosses are conspecific, and that the Canarian populations belong to this putative species, with only one taxon thus involved. *Orthotrichum shevockii* (the priority name) is therefore recognized as a morphologically variable species that exhibits a transcontinental disjunction between western North America and the Canary Islands. Within its distribution range, the area of occupancy is limited, a notable feature among bryophytes at the intraspecific level. To explain this disjunction, divergence time and ancestral area estimation analyses are carried out and further support the hypothesis of a long-distance dispersal event from California to Tenerife Island.

## Introduction

Since Wegener’s plate tectonics theory was proposed, ancient fragmentation has long been considered the main process explaining common distribution patterns in plant biogeography [1], while dispersal is seen as a random and irrelevant process [2]. However, more recent molecular tools and the development of dating and divergence time estimations have pointed to dispersal as a key process contributing to current species distributions [3–7]. In the case of oceanic islands, which originate without a former connection to a continental landmass, dispersal is considered to play a fundamental role in the generation of biodiversity and biogeographical patterns [2,8–12]. For the Macaronesian islands, a biogeographic region that encompasses the archipelagos of the Canary Islands, the Azores, Madeira and Cabo Verde (but see Vanderpoorten et al. 2007), it has been suggested that the endemic bryophyte component of the flora has a different biogeographical origin compared to angiosperms. This pattern has been explained, at least partially, by their different dispersal capabilities, since ancestors of a few endemic bryophytes seem to have colonized the islands from more distant continental pools [13,14]. Similarly, compared to tracheophytes, the larger distribution ranges of bryophytes have been attributed to their higher dispersal capabilities [15]. In many cases, these ranges involve intercontinental disjunctions at the species level, while in vascular plants these mostly occur at a genus level [15,16].

Recent studies have provided evidence for the traditional hypothesis that vicariance through ancient fragmentation may explain the origin of widely disjunct distributions in a few bryophyte lineages [17–19]. There is growing evidence that long-distance dispersal (LDD), however, has shaped the bulk of transoceanic bryophyte distributions [20–23]. This phenomenon also applies to taxa present in Macaronesia [24–26]. Despite this, it is worth noting that processes like incomplete lineage sorting, slow evolutionary rates [27–29], and cryptic speciation (for review see [30,31]), may contribute to establishing apparently disjunct distributions in bryophytes. This may also be the result of incomplete taxonomical knowledge [32,33]. All of these caveats call for accurate species delimitation methods, which are a necessary first step in assessing distribution patterns, and performing biogeographic analyses in widely distributed bryophytes. To this end, a plethora of integrative approaches has proved useful [23,29,32–38].

During recent field surveys on Tenerife Island (Canary Islands), several saxicolous populations of an unknown *Orthotrichum* Hedw. species were found in the area of El Teide National Park, at altitudes around 2100 m a.s.l., growing in protected crevices of volcanic rocks and walls. A preliminary morphological examination of these specimens revealed that their main characteristics differed from any *Orthotrichum* species known in the Mediterranean and North Atlantic areas. Surprisingly, these populations resembled two Californian species: *O*. *shevockii* Lewinsky-Haapasaari & D.H. Norris, and *O*. *kellmanii* 1D.H.Norris, Shevock & Goffinet. *Orthotrichum shevockii* is a saxicolous moss described from two localities in dry mountain areas in the southern Sierra Nevada, California, between 1150 and 1600 m a.s.l.; it is restricted to granitic rock outcrops, ceilings and underhangs of large boulders where plants receive only indirect sunlight and moisture by capillarity supply from the rock surfaces. *Orthotrichum kellmanii* is another saxicolous species, known from just a few localities in the coastal mountains of central California, where the climatic regime is characterized by high winter rainfall and infrequent summer fog from the Pacific Ocean. It grows on sandstone rock outcrops in chaparral areas, at altitudes around 650 m a.s.l. These two similar species mainly differ in gametophytic traits. *Orthotrichum shevockii* is characterized by leaves with bi- to tristratose margins and highly papillose leaf cells [39], whereas the leaves of *O*. *kellmanii* have a completely bistratose lamina [40]. However, the description of *O*. *kellmanii* based its diagnostic characters mainly on the presence of heterophyllous leaves (reproductive and vegetative stems leaves have different shapes), and a weakly cladocarpous growth across the substrate. The specimens from the Canary Islands have leaves with a highly variable degree of bistratosity among different individuals, from completely bistratose leaf laminae to bistratosity restricted to the leaf margins.

The bryophyte flora of Macaronesia is one of the best known among oceanic island regions worldwide [14]. However, the knowledge of its diversity and levels of endemism is still incomplete, as suggested by the increasing number of recent descriptions and re-circumscriptions of species for this region (*e*.*g*. [29,35,38,41,42]). Herein, in order to evaluate the true nature of the populations discovered in the Canary Islands, we address the following questions: (1) What is the identity of the new moss found in the Canary Islands? (2) What are the taxonomical relationships between the Canarian *Orthotrichum* moss lineages and the Californian *O*. *shevockii* and *O*. *kellmanii*? (3) What is the evolutionary and biogeographical history of these lineages? To accurately address these questions, we used an integrative taxonomic approach combining morphological analyses, phylogenetic inferences, molecular dating and the estimation of ancestral ranges.

## Material and methods

Field collecting permits were granted by both California state and federal ownership, where applicable, as well as from Parque Nacional del Teide.

### Sampling design

**Fig 1:**
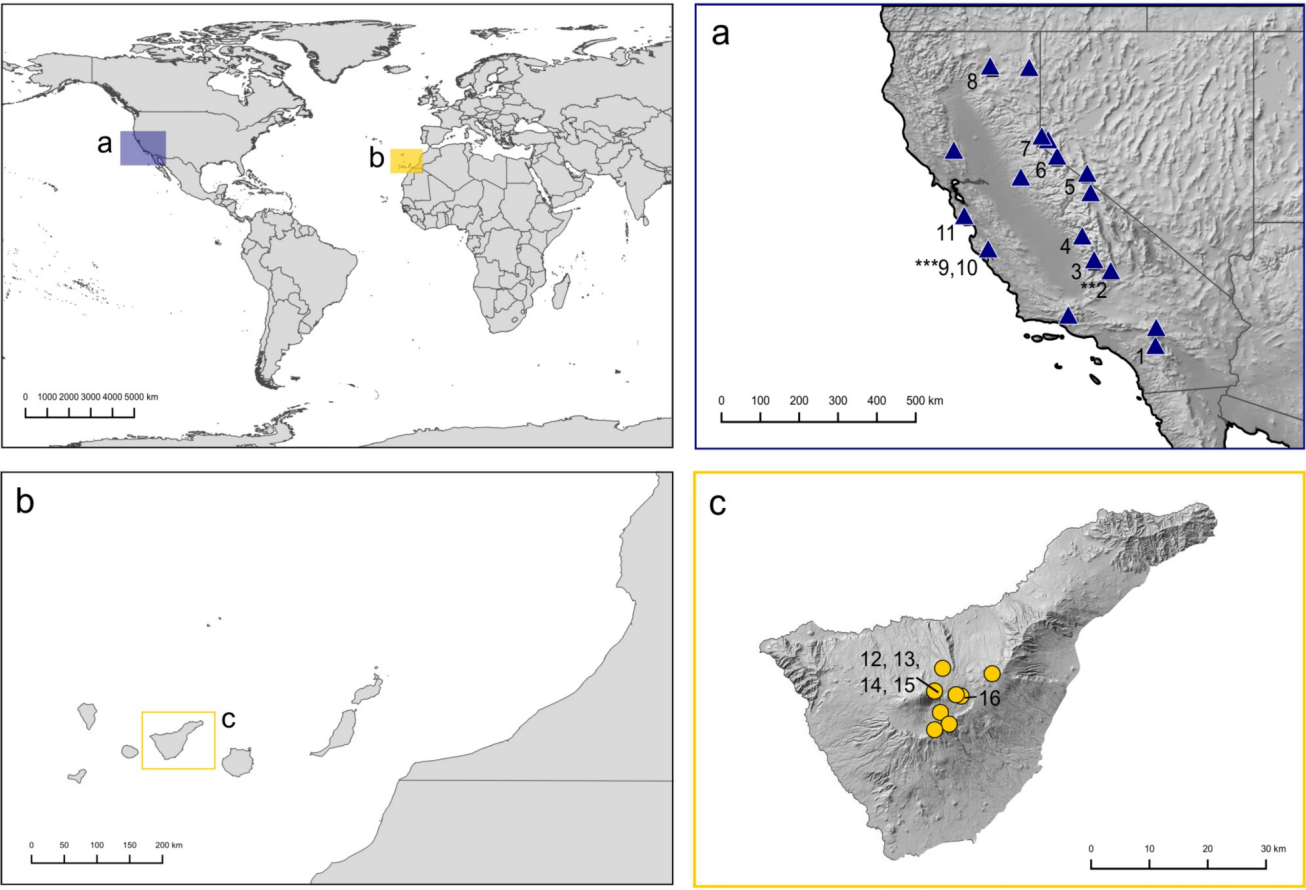
Geographic origin of the studied specimens. (a) California and Nevada, (b) the Canary Islands, (c) Tenerife. Numbers indicate specimens included both in morphometric and phylogenetic analyses (see S1 and S2 Appendices). ** = original locality of *O*. *shevockii*, *** = original locality of *O*. *kellmanii*.

The material for this study includes the collections sampled on Tenerife, herbarium specimens of *O*. *shevockii* and *O*. *kellmanii* from UC, CAS, CONN and NY herbaria (including type material), and specimens obtained during specific collecting campaigns to several Californian mountain ranges and neighboring regions of western Nevada. Specimens were selected to represent the whole distribution and ecological range of the species. For the target species, 30 samples were included in the morphological analyses: nine from Tenerife and 21 from California (Fig 1 and S1 Appendix). Based on the availability and quality of the specimens, a subset of 16 samples representative of the morphological diversity and geographic distribution of the species was selected for molecular analyses (Fig 1 and S2 Appendix). To provide a phylogenetic context for the assessment of the monophyly in these two putative species, along with the populations from the Canary Islands, in addition to our ingroup sequences, we included specimens of other *Orthotrichum* species from the western coast of North America and the Canary Islands, some endemic to these areas [25,33,39]. Three species of *Lewinskya* F.Lara, Garilleti & Goffinet, one of *Macrocoma* (Hornsch. *ex* Müll.Hal.) Grout, one of *Nyholmiella* Holmen & E.Warncke, and two of *Zygodon* Hook. & Taylor were selected as outgroups, resulting in a total of 66 samples (see S2 Appendix for voucher information and GenBank accession numbers).

### Morphological analyses

A morphological analysis was conducted on the 30 selected specimens to assess the differences between the Californian plants attributed to either *O*. *shevockii* or *O*. *kellmanii*, and those from Tenerife. A set of morphological characters, both qualitative and quantitative, was selected and studied according to our previous experience with Orthotrichaceae [32,33,43–45]. Qualitative traits of the gametophyte included plant habit and several leaf characters such as leaf shape, leaf margin, lamina bistratosity, and cell papillosity. Sporophyte characters are usually of great diagnostic value for the genus [46,47], and we therefore focused the study on capsule shape, exothecial band structure, stomata position, structure and ornamentation of the peristome calyptra, and vaginula hairiness.

For quantitative morphometric analyses, 16 characters were selected. Measurements and construction of the dataset protocol followed Vigalondo et al. [45]. To detect a possible unknown underlying structure within the dataset, an exploratory multivariate analysis was performed (principal component analysis, PCA). A correlation matrix was used in the PCA to scale the morphological variables, and only principal components (PCs) accounting for more than 10% of the variance were considered in the results. Univariate analysis of variance (ANOVA) was conducted to assess the homogeneity of variances for each of the 16 quantitative variables for California and Tenerife specimens. Multivariate analyses were run twice: (i) discarding samples with missing values; and (ii) replacing missing values with the mean value of each character. Results from the two approaches were congruent, so to avoid reducing the sample size, we used the data set with missing values replaced with the mean for the final analyses (S1 Table). Descriptive statistics were ultimately computed for all quantitative variables, considering populations from Tenerife and California separately. The results were summarized in the form of beanplot graphs [48], representing the empirical density shape, mean, and all individual observations for each of the two evaluated geographical groups. All statistical analyses were conducted in R v.3.3.1 [49].

### DNA extraction and sequencing

DNA was extracted from apices of stems and branches from dried herbarium specimens using the DNeasy Plant Mini Kit for DNA isolation (Qiagen). We selected four loci previously used for phylogenetic reconstructions of *Orthotrichum* [33,45]: three chloroplast loci, namely *atp*B-*rbc*L, *rps*4, and *trn*L-F, and the nuclear internal transcribed spacer II (ITS2). The primer pairs used for each locus were *atb*1/*rbc*L1 [50], *rps*A/*trna*S [51,52], *trn*C/*trn*F [53] and *ITS*2*F/ITS*2*R* [54].

Double-stranded DNA templates were prepared by PCR, which was performed using Ready-To-Go PCR Beads (Amersham Pharmacia Biotech Inc.) in a final reaction volume of 25 μL according to the manufacturer’s instructions. PCR amplification of *atp*B-*rbc*L, *rps*4, and *trn*L-F was performed using the protocol described in [33], while the ITS2 protocol followed [45]. PCR products were purified using the Exo/SAP protocol (Thermo Fisher Scientific, Spain). Samples were incubated with 1 μL of Exo1 enzyme and 4 μL of FastAP following the manufacturer’s instructions. Cleaned PCR products were sequenced by Macrogen (www.macrogen.com). All new sequences were deposited in GenBank (see S2 Appendix).

### Phylogenetic and dating analyses

Nucleotide sequence contigs were edited and assembled for each DNA region using Geneious 7.1.2 (http://www.geneious.com, [55]) and PhyDE v.0.9971 [56]. Sequences were aligned manually and trimmed at the ends. Regions of ambiguous or incomplete data were identified with GBlocks [57] and excluded from subsequent analyses.

Phylogenetic analyses were performed using maximum likelihood (ML) and Bayesian inference (BI). The best-fitting substitution models for each locus were inferred under the Bayesian Information Criterion (BIC) in jModelTest v.2.1.3 [58]. Maximum likelihood analyses were run with RAxML 8 [59], and the best ML tree was selected from 100 iterations and its support was assessed with 1000 replicates of bootstrap resampling under the ML criterion. Bayesian phylogenetic analyses were carried out using MrBayes v.3.2.1 [60]. The Markov chain Monte Carlo (MCMC) was run for 2 to 5 million generations with two runs and four chains, sampling trees, and parameters every 1000 generations. After checking that stationarity had been reached (*i*.*e*. the average standard deviation of split frequencies remained below 0.01 for the last 10,000 generations), posterior probabilities (PP) were estimated from the 50% majority-rule consensus trees after a burn-in of 25% of the starting trees. The resulting trees for both ML and BI analyses were plotted using FigTree v.1.4.2 [61].

Insertions and deletions (indels) in non-coding regions are sometimes difficult to assess [62] and can lead to ambiguous alignments. To determine the effect of their inclusion, phylogenetic information from indels was coded as an adjacent block with the program SeqState [63], using the simple indel coding method [64]. The analyses were performed with and without codified indels with the same parameters indicated above, using model F81 for the indel partition in MrBayes, as recommended by [65]. The inclusion of the indels did not change the topology of the trees or result in increased statistical support as measured by PP, and further analyses were performed on the matrix treating the indels as missing data.

All independent gene data sets were combined in a single concatenated matrix, as no incongruences were identified in branches supported with posterior probability ≥ 0.95 and bootstrap support ≥ 85 when each gene was analyzed separately. The final analyses included only those sequences for which all loci were obtained, thus discarding 11 sequences from the final matrix. The resulting concatenated data set was analyzed in PartitionFinder [66] to select the best partitioning scheme and nucleotide substitution model, using the greedy algorithm with linked branch lengths under the BIC criterion. Three partitions were defined: ITS2 (HKY+G), *rps*4 (HKY+G) and the combined *atp*B-*rbc*L and *trn*L-F (GTR+G).

Divergence times were estimated using BEAST 1.8.0 [67]. Because the inclusion of identical sequences in dating analysis results in many zero-length branches at the tip of the tree and can cause the model to over partition the dataset [68], we reduced the data set to haplotypes (30 sequences) using DnaSP 5.10.1 [69]. This program considered all samples from the Canary Islands as a unique haplotype, although one of them appeared outside the main group of this area in the phylogenetic analyses. Thus, we compared BEAST analyses with the sample (BV050 [13]) either separate or included in the general haplotype from the Canary Islands. For all the analyses, clock and tree models were linked across partitions, and models of substitution were unlinked across the three partitions. Both strict and uncorrelated log-normal relaxed clocks were tested under two different speciation tree models: Yule and birth-death process. In the absence of fossil records of *Orthotrichum*, the uncertainty of dating estimates was modelled with a uniform distribution under two increasingly conservative nucleotide substitution rate assumptions incorporated into the *ucld*.*mean* parameter in BEAST. First (analysis I), we used an absolute substitution rate of mean = 4.453E^-4^ and stdev = 1.773E^-6^ substitutions/site/million years, inferred from relaxed-clock analyses across the Moss Tree of Life [70]. Second (analysis II), to provide an alternative to time estimates that might be overestimated (given that Laenen et al. [70] performed the analyses at the generic level), we applied a distinct rate for the plastid (5.0E^-4^, stdev range 2–8E^-4^, subst./site/ma) and nuclear partitions (4.13E^-3^, stdev range 1.72–8.34E^-3^, subst./site/ma), proposed by Villarreal & Renner [71]. All BEAST analyses were run for four independent chains of 40 million generations each, sampling every 10 thousand generations and their convergence was assessed by confirming that all parameters had reached stationarity and sufficient effective sample sizes (> 200) in all converged runs using Tracer v1.6 [72]. The best model was selected through marginal likelihood estimates (MLEs) that were assessed using path-sampling (PS, [73]) and stepping-stone (SS, [74]) methods. The resulting MLEs were averaged across replicate runs to generate a single PS and SS value for each model. The obtained MLEs for all hypotheses were ranked, and Bayes factors were then calculated. In this study, the birth-death process model performed best (S2 Table). After discarding the burn-in steps, tree files from the four independent runs of the selected model were combined using LogCombiner 1.8 [67] and the resulting maximum clade credibility (MCC) tree was summarized in TreeAnnotator 1.8 [67] and viewed in FigTree v.1.4.2 [61].

### Ancestral area estimation

To infer the historical biogeography of *O*. *shevockii*, we defined six geographical areas, also considering the whole distribution of the rest of the ingroup species: western North America (W), eastern North America (E), Caribbean, Central America and South America (N), Europe (U, including the Mediterranean and North Africa), Macaronesia (M), and Asia (A). We used the time-calibrated MCC tree obtained from BEAST, but removing the outgroups, to perform ancestral area estimations across the *Orthotrichum* ingroup with the R package BioGeoBEARS [75]. BioGeoBEARS allows the use of the Lagrange DEC model (Dispersal-Extinction-Cladogenesis), which includes dispersal (*d*) and extinction (*e*) as free parameters, and a model (DEC+J) that includes an additional parameter J taking founder-event speciation into account ([75] and references therein). Since different ancestral area reconstructions are based on different assumptions, one can compare these two versions of the DEC model with a likelihood version of the Dispersal-Vicariance Analysis (DIVALIKE), and a likelihood version of the range evolution model of the Bayesian Binary Model (BAYAREA), with the option of also adding founder-event speciation to either of these two alternative models. However, in a recent study, Ree and Sanmartín [76] proposed that DEC+J might be a poor model of founder‐event speciation and statistical comparisons of its likelihood with a pure DEC model may be inappropriate. Consequently, we refrained from implementing the DEC+J in the present study and focused on the classical versions of the three biogeographical models implemented in BioGeoBEARS (DEC, DIVALIKE, BAYAREA). These three models were estimated under a maximum likelihood framework, and compared in terms of how well they fitted the data using the Akaike Information Criterion (AIC) [75,77]. Implementing the same best-fit model of ancestral area estimation (i.e. DEC; see results section), we determined the degree to which differences in tree topology and branch lengths yield different ancestral area estimations for nodes by using a customized script to run BioGeoBEARS on a subset of 100 BEAST trees randomly sampled from the posterior probability distribution obtained from BEAST analyses.

## Results

### Morphology

**Fig 2:**
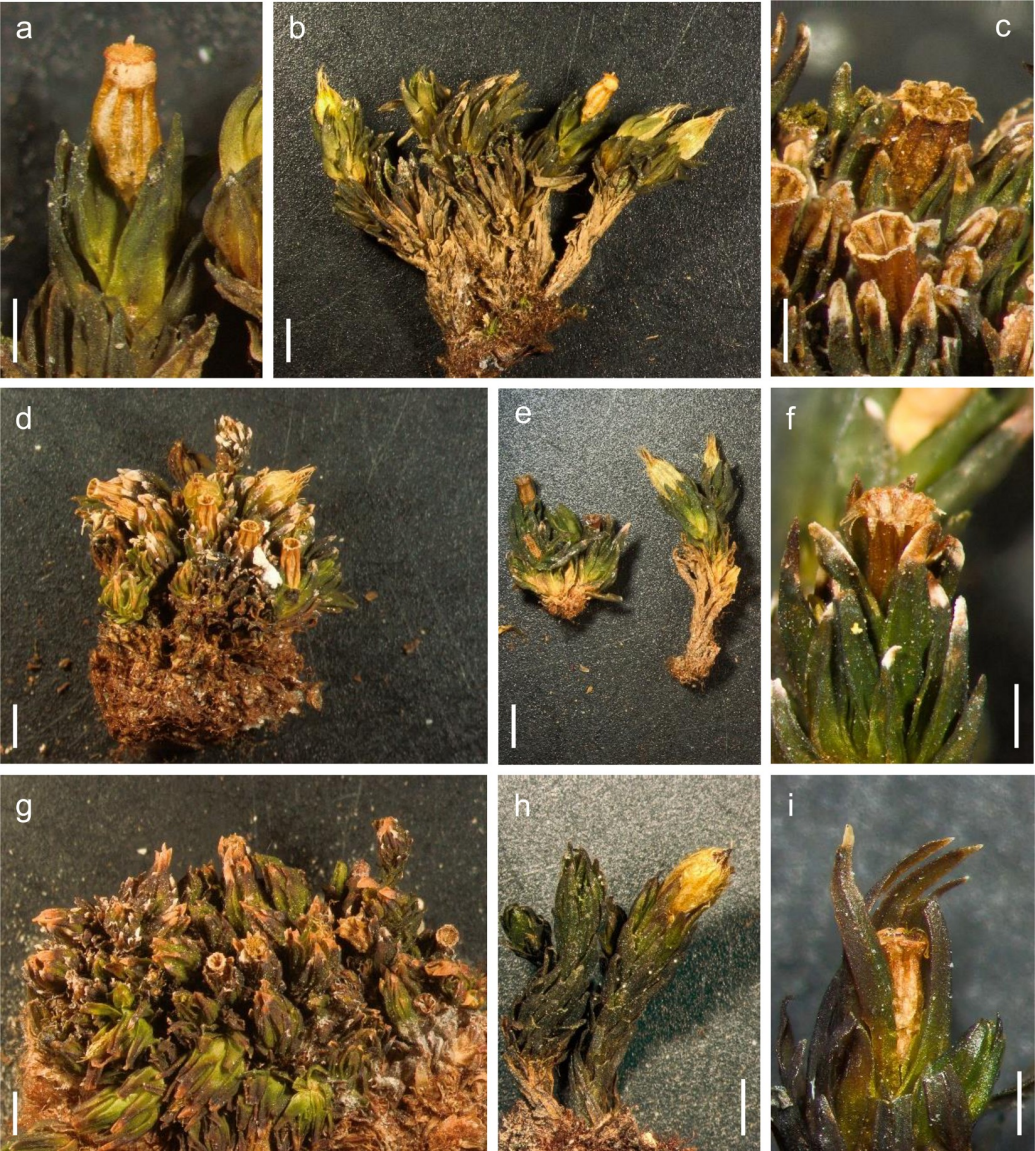
Plant habit and dry capsule characteristics of specimens from the different geographic areas. (a-d) *Orthotrichum shevockii* from Western North America; (e-g) samples from Tenerife; (h-i) *O*. *kellmanii* from California. (a, c, f and i) capsule detail; (b, d, e, g and h) habit; (e) two different habits from the same voucher. Scale bars: a, c, e and i = 0.5 mm, b, d, f, g and h = 1 mm. Vouchers: a-b, *Shevock 13404* (CAS 958716, paratype); c, *Shevock 21948* (CAS 1040048); d, *Shevock 21802* (UC 1754431); f, *Losada-Lima*, *León & Díaz s*.*n*. (TFC-Bry 15904); e-g, *Losada-Lima s*.*n*. (TFC-Bry 17428); h, *Shevock 32935* (MAUAM 5097); i, *Shevock* 32935 (NY 1140598).

**Fig 3:**
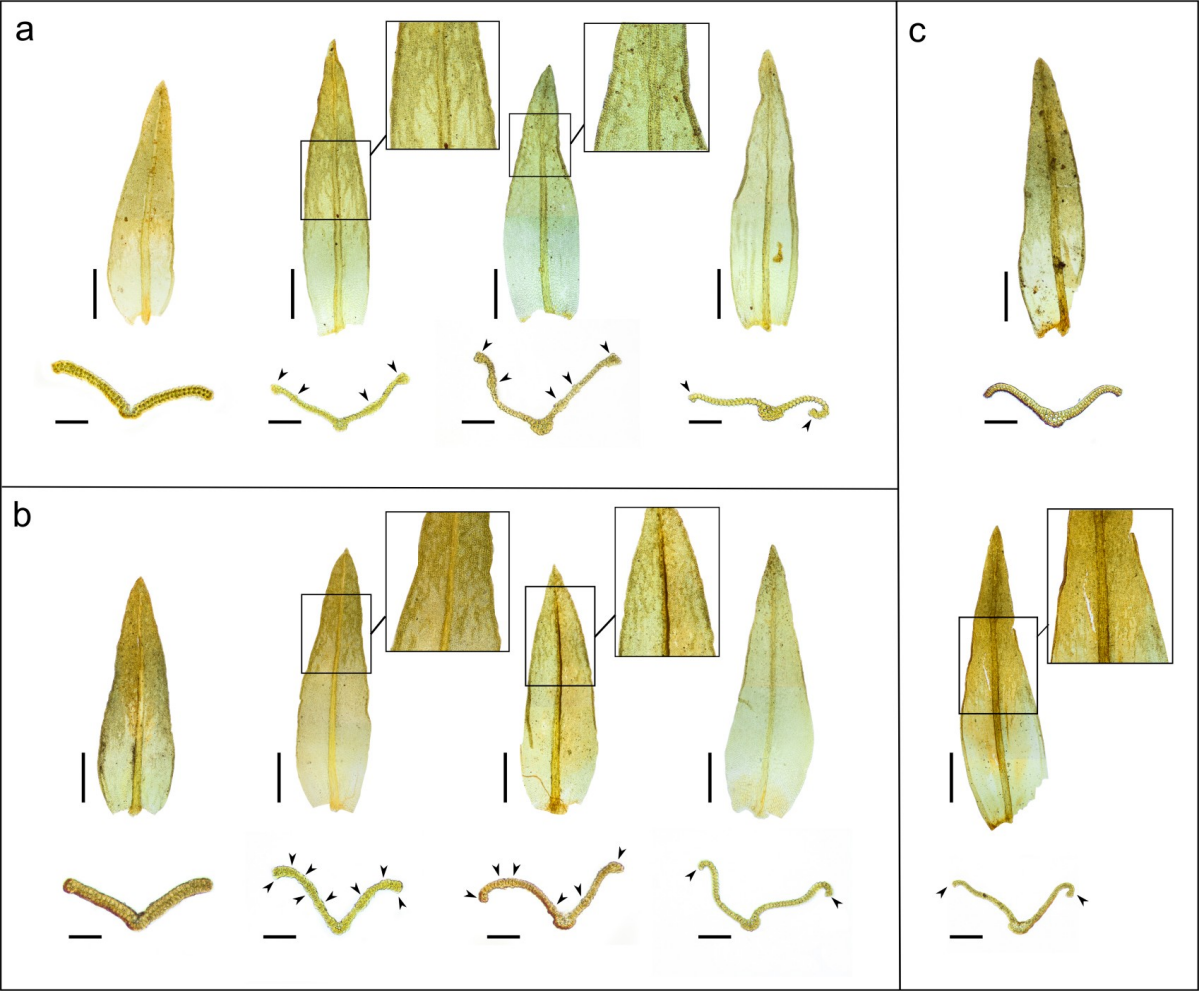
Leaf thickness variation and leaf cross-sections of specimens from the different geographic areas. (a) *Orthotrichum shevockii* from western North America; (b) samples from Tenerife, (c) *O*. *kellmanii* from California. (a,b) from left to right: bistratose leaf (except base), leaf with a bistratose upper part and bistratose bands, leaf with dispersed bistratose bands in the upper part, leaf almost unistratose with bistratose patches around the apex. (c) top: bistratose leaf (except base), bottom: leaf with bistratose upper part and bistratose bands. Each leaf belongs to a separate individual. Cross-sections belong to a different leaf of the same individual. Arrowheads indicate bistratose strands or margins; in (c) they indicate the tristratose margins. Scale bars: leaves = 0.5 mm, cross sections = 100 μm. Vouchers: a, *Shevock & Anderson 16754* (UC 1754230), *Lara*, et al. *s*.*n*. (MAUAM-Brio 3289), *Shevock & York 13404* (CAS 958716, paratype of *O*. *shevockii*), *Shevock 21802* (UC 1754431); b, *J*.*M*.*B*., *J*.*G*.*M*. *& J*.*L*.*P s*.*n*., (TFC-Bry 15957), *Losada-Lima s*.*n*. (TFCBry-17428), *Losada-Lima*, *León & Díaz s*.*n*. (TFC-Bry 15861), *Losada-Lima*, *León & Díaz s*.*n*. (TFC-Bry 15904); c, *Shevock 32935* (NY 1140598).

**Fig 4:**
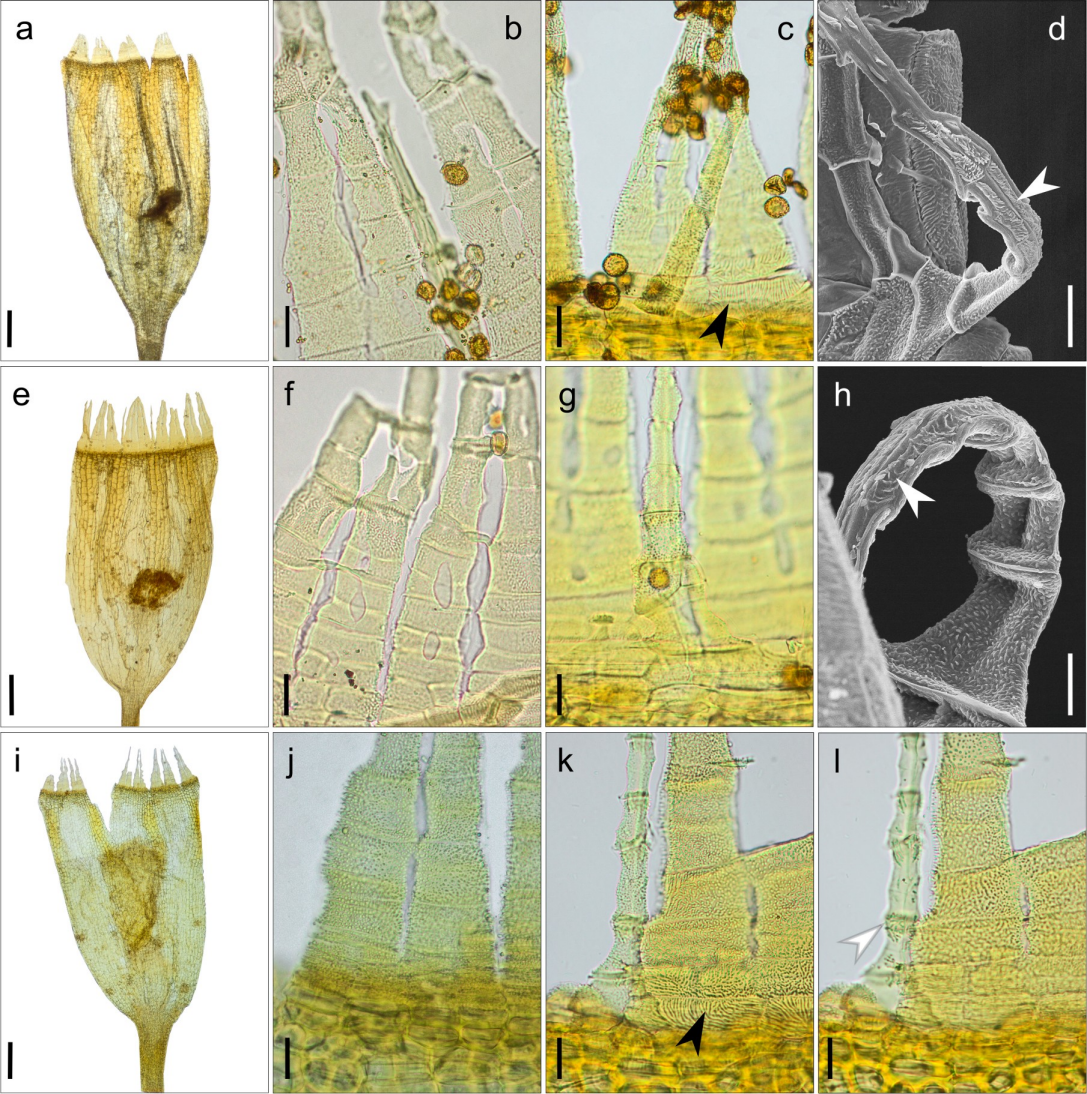
Capsule and peristome ornamentation of specimens from different geographic areas. (a-d) *Orthotrichum shevockii* from western North America; (e-h) samples from Tenerife; (i-k) *O*. *kellmanii* from California. (a, e and, i) capsules with stomata mostly restricted to the neck; (b, f and j): exostome structure, showing the teeth lacunosity; (c, g and k) endostome internal layer (IPL) papillose ornamentation; (c and k) exostome internal layer (PPL), black arrows indicate striae at base; (d, h and l) endostome external layer (PPL) ornamentation; white arrows indicate lines or striae, sometimes forming plaques. Scale bars: a, e and i = 200 μm; b-c, f-g, j-l = 20 μm; d-h = 10 μm. Vouchers: a, *Lara*, et al. *s*.*n*. (MAUAM-Brio 3289); b, d, *Shevock 13404* (CAS 958716, paratype), c, *Shevock 21802* (UC 1754431); e, *Losada-Lima s*.*n*. (TFC-Bry 15567); f, *Losada-Lima*, *León & Díaz s*.*n*. (TFC-Bry 15904); g, *Losada-Lima s*.*n*. (TFC-Bry 17406); h, *Losada-Lima*, *León & Díaz s*.*n*. (TFC-Bry 15952); i-j, *Shevock 32935* (MAUAM 5097); k-l, *Shevock 32935* (NY 1140598).

Only a limited number of specimens from California and Nevada could be clearly ascribed to either *O*. *shevockii* or *O*. *kellmanii*. This was not possible for the majority of specimens, which actually showed morphological traits of both taxa, traits that can also display a wide range of variation (Figs 2A–2D and 3H–3I). Moreover, all Californian specimens share some characteristics that are very uncommon in the genus *Orthotrichum*, such as: (1) stomata that appear restricted to the capsule neck, and only occasionally reach the base of the urn (Fig 4A and 4I); (2) exostome teeth that are usually lacunose, showing lacunae in both the external and internal layers around the median line, sometimes appearing within the teeth cell areas (Fig 4B, 4J and 4K); and (3) an endostome external layer that is frequently ornamented with oblique or vertical lines (Fig 4D and 4L).

Other qualitative characters exhibit a wide range of variation among and within regions, and frequently even among stems from the same cushion. Plants commonly form short and dense cushions or tufts, but in some cases, they look longer and looser (Fig 2B and 2D). Concerning gametophyte traits, those related to leaves show great variation (Fig 3A). Leaf margins are typically bistratose for most of their length, occasionally 3–4 cells thick and rarely unistratose. Similarly, the leaf lamina is predominantly or completely bistratose in its upper 1/2-2/3 part, but can have sparse bistratose bands in its upper half or, rarely, only small bistratose strands or patches restricted to the apex. Papillosity of leaf lamina is also highly variable, with cells featuring 2(3–4) papillae on each side; papillae can be prominent, simple or bifurcate, or in other cases short or even negligible. Papillosity to a great extent appears to be related to leaf thickness: bistratose leaves have lamina cells with low papillae or almost smooth, while leaves only bistratose at the margins or with bistratose strands in the lamina show high and bifurcate papillae. Upper leaves, both vegetative and perichaetial, can be broadly to narrowly lanceolate, and their apices are usually acute, although sometimes perichaetial leaves are shortly acuminate (Fig 3A).

As for the sporophytic traits, capsule exothecial bands are formed by 4–8 isodiametric to rectangular cells, varying among samples, and usually extend along the whole urn length, but are occasionally restricted to the upper half only (Fig 4A). The ornamentation of the different components of the peristome is noticeably variable (Fig 4B–4D). The exostome outer layer consists of a reticulum at the basal part, where transverse lines are usually more apparent, and it is covered by a variable proportion of papillae. In contrast, the middle and upper parts of teeth have a denser ornamentation with a predominance of tall (occasionally low) papillae or, less frequently, vertical lines (Fig 4B). Ornamentation of the inner surface of the exostome can be papillose, reticulate, striate, or a mix, and predominantly shows very well-marked vertical striae at the base (Fig 4C). Regarding the ornamentation of the endostome, the internal layer is rugulose or papillose, with papillae sometimes densely disposed and variably prominent (Fig 4C), while the external layer can be smooth or variably ornamented with lines, sometimes densely grouped in plaques (Fig 4D).

The isotype material of *O*. *kellmanii* and two other samples originally ascribed to this species fit the variability encountered in the rest of the Californian samples (Figs 2H and 2I and 3C and 4I–4L). They also exhibit the above-mentioned characteristics. The only peculiarities noticed for these three samples concern leaf characteristics. As in other Californian samples, leaves are extensively bistratose, both in margins and lamina, but exceptionally show up to three layers of cells in areas adjacent to the nerve and near the apex (Fig 3C). Additionally, the perichaetial leaves are consistently shortly acuminate. Although most sporophytes show the typical structure described above, in some capsules the exothecial bands are unusually weak, made up of 3–6 cell rows and restricted to the upper part of the urn (Fig 4I). None of the material shows a cladocarpous growth pattern as described in Norris et al. [40].

The specimens from both Tenerife and California share all the qualitative characters mentioned above, and exhibit the same degree of variation for gametophytic (Figs 2 and 3) and sporophytic traits (Fig 4). The few peculiarities observed affect only the frequency of some leaf traits. In Tenerife, the leaf lamina of most samples is commonly partially bistratose, with bistratose margins and dispersed bistratose bands in the upper part of the lamina. Leaf cell papillae are commonly short, and perichaetial leaves are usually broadly lanceolate (Fig 3B).

**Fig 5:**
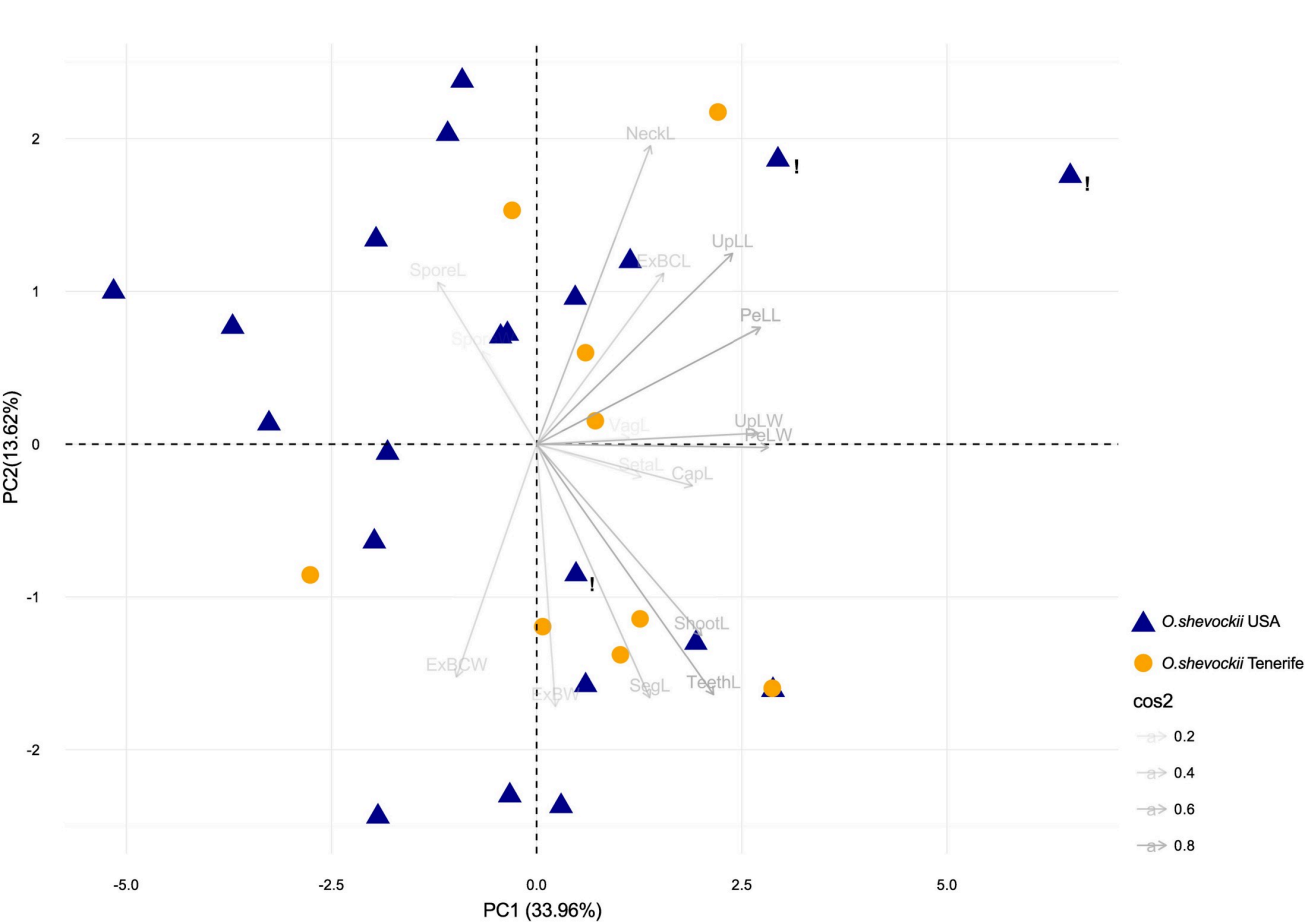
Principal component analysis (PCA) representing the first two components. The percentage of variance explained by each component is given between brackets. Arrows represent the variables included in the analyses. cos2 represents the squared loadings for variables. ! = samples originally identified as *Orthotrichum kellmanii*.

**Fig 6:**
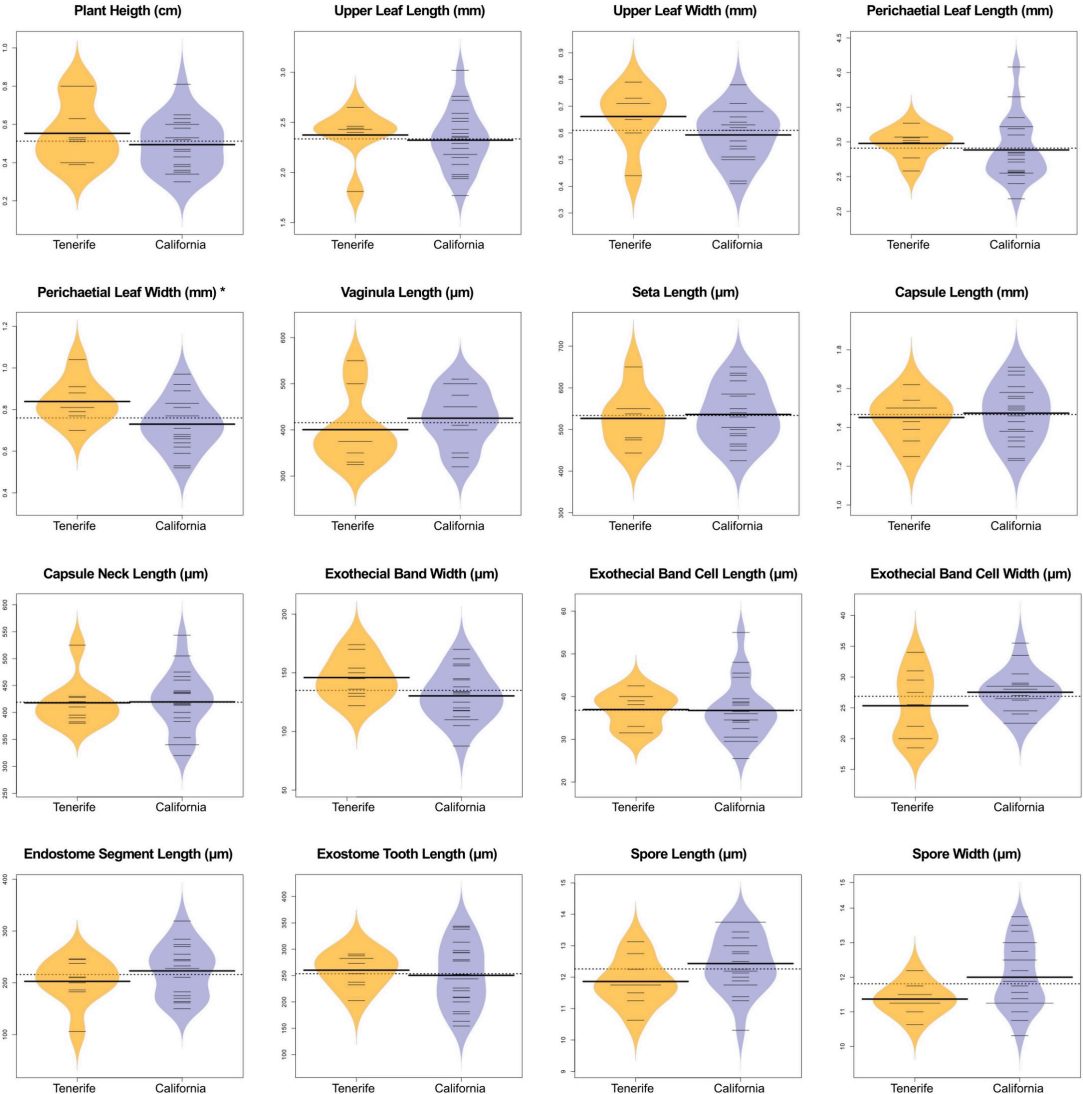
Beanplots of the quantitative variables for *Orthotrichum shevockii*. Yellow = Tenerife (Canary Islands), blue = California (western North America, including *O*. *kellmanii* isotype materials). Individual observations are represented by small horizontal lines (in the case of multiple observations with the same values, the corresponding number of lines were merged), mean per group is shown by a bold long line and the mean for all data by a dotted line. Estimated density of the data distribution is displayed by the density shape in grey (for details see [48]). Stars indicate ANOVA significance values: * 0.05.

Statistical analyses of morphological quantitative traits also showed no differences between California and Tenerife specimens. In PCA analyses, the three first principal components (PCs) accounted for 59.98% of the variance. The PCA biplot shows a dispersion of samples within the represented space, where specimens from California and Tenerife overlap without any geographical or taxonomical structure (Figs 5 and S1). With respect to the specimens originally identified as *O*. *kellmanii*, only one of them appears separated in the positive extreme of PC1. The most important variables in each of the three PCs are indicated in Table 1. When variables are considered independently comparing California and Tenerife, ANOVA analysis only shows significant differences for one variable: perichaetial leaf width (Table 1 and Fig 6).

**Table 1:** Quantitative characters evaluated for specimens of each geographic region, and results of quantitative morphometric analyses.

	Descriptive statistics ^a^			PCA^b^			
Character	Tenerife (CI)	*O*. *shevockii* California & Nevada (wNAm)	*O*. *kellmanii* California (wNAm)	PC1	PC2	PC3	ANOVA^c^
**Gametophyte**							
Shoot length ^1^	0.55 ± 0.16 [0.39–0.8]	0.48 ± 0.13 [0.3–0.81]	0.63 ± 0.04 [0.6–0.65]	0.277	-0.272	0.082	0.990
Upper leaf length ^2^	2.37 ± 0.26 [1.81–2.65]	2.27 ± 0.25 [1.77–2.72]	2.65 ± 0.43 [2.18–3.02]	0.329	0.272	-0.020	0.153
Upper leaf width ^2^	0.66 ± 0.11 [0.44–0.79]	0.57 ± 0.09 [0.41–0.71]	0.71 ± 0.06 [0.66–0.78]	0.372	0.016	0.072	2.212
Perichaetial leaf length ^2^	2.98 ± 0.21 [2.58–3.27]	2.79 ± 0.32 [2.18–3.35]	3.43 ± 0.78 [2.56–4.08]	0.376	0.166	0.081	0.306
Perichaetial leaf width ^2^	0.84 ± 0.1 [0.7–1.04]	0.7 ± 0.1 [0.52–0.89]	0.91 ± 0.07 [0.83–0.97]	0.389	-0.004	0.042	4.833*
**Sporophyte**							
Vaginula length	400.56 ± 76.46 [325–550]	423.33 ± 63.54 [320–510]	437.5 ± 53.03 [400–475]	0.155	0.010	0.054	0.866
Seta length	526.55 ± 68.57 [443.33–650]	521.76 ± 60.34 [425–635]	617.22 ± 32.5 [585–650]	0.175	-0.047	0.057	0.097
Capsule length ^1^	1.45 ± 0.11 [1.25–1.62]	1.46 ± 0.14 [1.23–1.69]	1.56 ± 0.17 [1.38–1.71]	0.261	-0.059	0.314	0.172
Capsule neck length	417.83 ± 44.41 [380–525]	410.42 ± 49.68 [320–505]	501.67 ± 58.92 [460–543.33]	0.191	0.425	0.053	0.007
Exotecial band width	145.94 ± 17.87 [122–174]	133.66 ± 18.9 [105–170]	110.5 ± 23.25 [87.5–134]	0.031	-0.374	0.132	3.869
Exotecial band cell length	36.96 ± 4.03 [31.5–42.5]	35.2 ± 5.56 [25.5–48]	46 ± 8.35 [38.5–55]	0.213	0.244	-0.181	0.007
Exotecial band cell width	25.33 ± 5.52 [18.5–34]	27.81 ± 3.05 [22.5–35.5]	25.83 ± 4.16 [22.5–30.5]	-0.135	-0.331	0.217	1.896
Endostome segment length	202.72 ± 43.38 [106–246.25]	213.69 ± 49.9 [150–319.38]	266.25 ± 9.92 [255–273.75]	0.190	-0.361	0.151	1.155
Exostome teeh length	260.27 ± 30.62 [202.5–290.83]	243.57 ± 59.54 [154.17–341.88]	291.93 ± 47 [252.04–343.75]	0.297	-0.356	-0.036	0.216
Spore length	11.86 ± 0.76 [10.63–13.13]	12.47 ± 0.94 [10.31–13.75]	12.19 ± 0.81 [11.38–13]	-0.166	0.231	0.574	2.722
Spore width	11.37 ± 0.44 [10.63–12.19]	12.03 ± 1.01 [10.31–13.75]	11.85 ± 0.79 [11.25–12.75]	-0.091	0.133	0.648	3.512

^a^ Descriptive statistics (mean ± SD [range]); all measurements are in μm except those with ^1^ = cm and ^2^ = mm.

^b^ PCA component loadings for each original variable are represented, in bold variables with the highest loadings for each component; percent of total variance explained for first component (PC1) = 33.96%, PC2 = 13.62% and PC3 = 12.4% (see Fig 5 and S1 Fig).

^c^ ANOVA F statistic and significance level (* ≤ 0.05) for each variable for Canary Islands and western North America (including the three samples of *O*. *kellmanii* = *O*. *shevockii*). CI = Canary Islands, wNAm = western North America.

### Phylogeny, dating and ancestral area reconstruction

**Fig 7:**
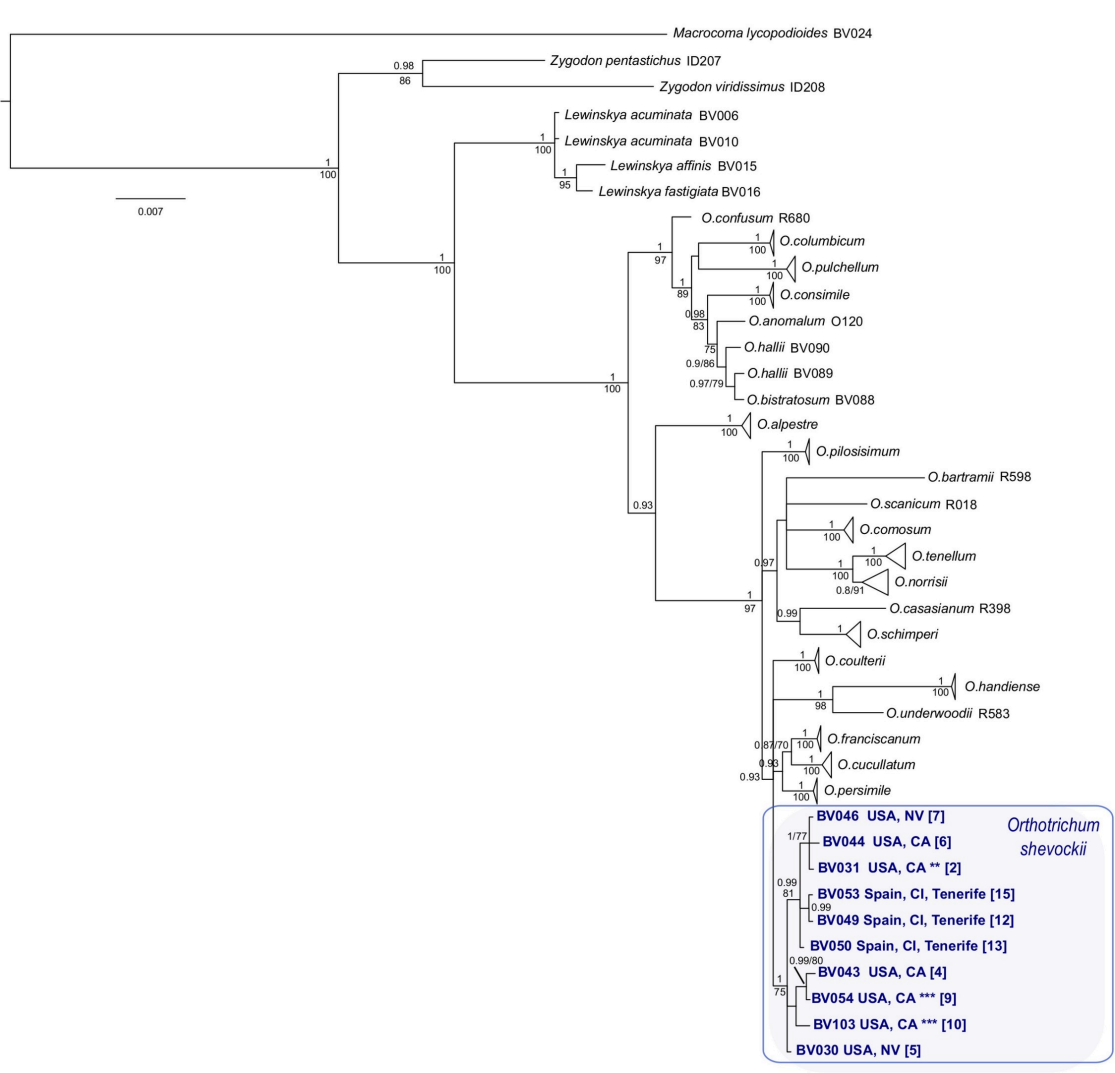
Majority-rule consensus tree obtained in the Bayesian analysis. Bayesian posterior probabilities (≥ 0.90) and maximum likelihood bootstrap values (≥ 70%) are shown above and below branches, respectively. Sequence labels of *Orthotrichum shevockii* are followed by identification number, geographical origin, and number identification between brackets as in Fig 1 and S1 and S2 Appendices. ** = paratype material of *O*. *shevockii*, *** = isotype material of *O*. *kellmanii*.

Information regarding sequence length and variability within each marker and the combined matrix is presented in Table 2, whereas pairwise differences among ingroup samples are shown in S3 Table. Phylogenetic analyses of ML and BI (Fig 7) group samples from California and Tenerife in the same monophyletic lineage with high support (BS = 75, PP = 1.0). This lineage is embedded within a clade with a PP of 0.93, composed of taxa restricted to California and Nevada along with *Orthotrichum handiense* F.Lara, Garilleti & Mazimpaka from Fuerteventura (Canary Islands). Samples of *O*. *kellmanii* are also placed within the clade of *O*. *shevockii* in BI and ML analyses.

**Table 2:** Characteristics of the four sequenced DNA regions and the resulting combined matrix used for phylogenetic analyses.

	*ITS*2	*rps*4	*trn*L-F	*atp*B-*rbc*L	Combined
Sequences	60	66	64	63	55
Aligned length (bp)	543	642	508	560	1715
Total matrix					
Variable sites	184	98	132	124	317
Potentially informative sites	72	62	114	87	243
*Orthotrichum shevockii*					
Variable sites	4	1	5	6	12
Potentially informative sites	4	1	3	6	11
Substitution model (BIC)	HKY+G	HKY+G	HKY+G	GTR+G	

**Fig 8:**
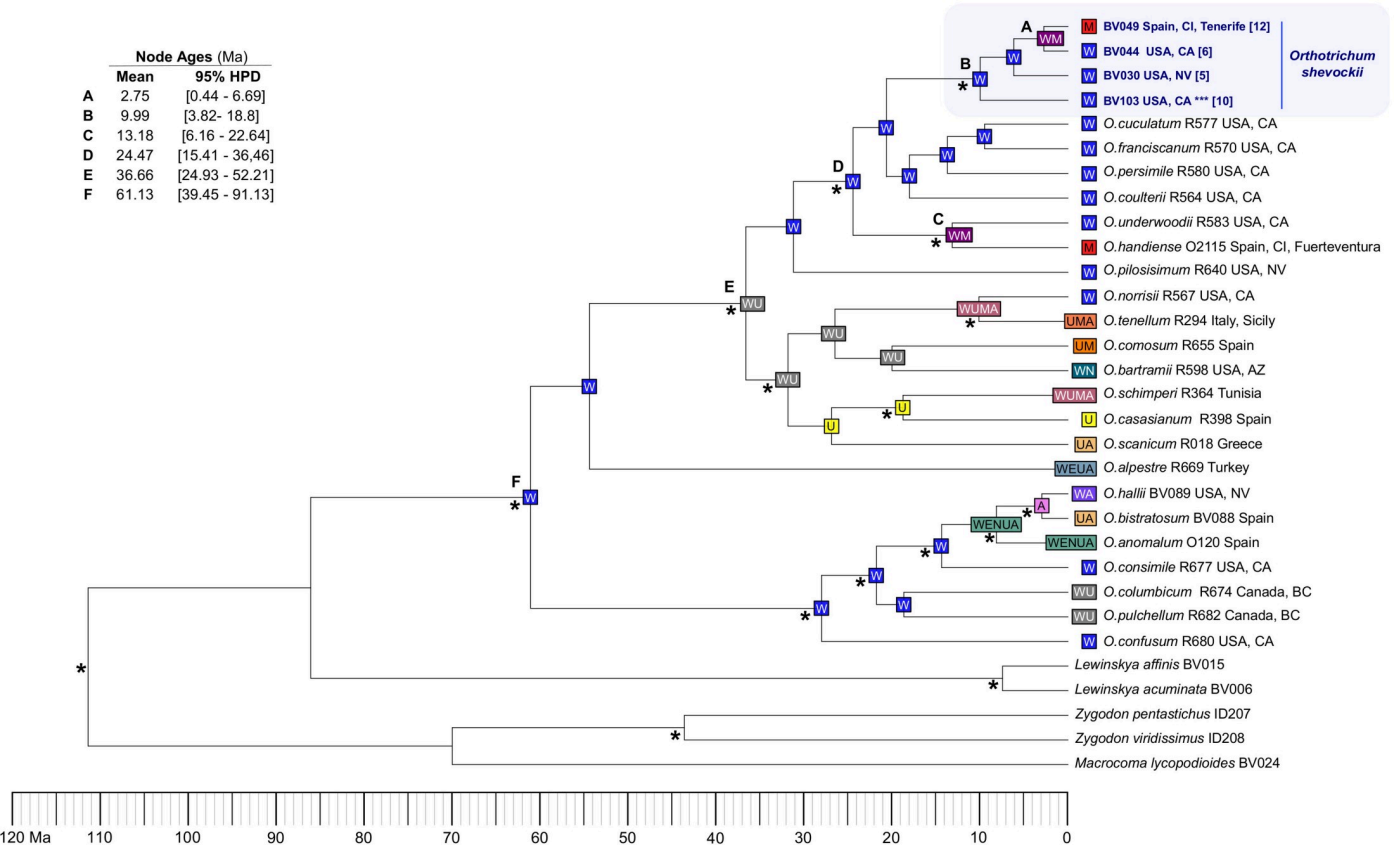
Molecular dating and biogeographic analyses. Maximum clade credibility tree from the relaxed molecular-clock analysis of the four loci in BEAST with an absolute nucleotide substitution rate (analysis I, mean = 4.453E^-4^, stdev = 1.773E^-6^subst./site/ma). Asterisks (*) at nodes refer to highly supported nodes (PP >0.95). Sequence labels are followed by identification number and geographical origin. In the case of *Orthotrichum shevockii*, sequence labels are also followed by an identification number between brackets as in Fig 1 and S2 Appendix (*** = isotype material of *O*. *kellmanii*). For the *Orthotrichum* group, letters in colored boxes in each node show the area or combination of areas with the highest probability of being ancestral, according to the reconstructions based on the DEC model implemented in BioGeoBEARS. Letters correspond to the following ancestral areas or combination of areas: W = western North America; E = eastern North America; N = Neotropics; M = Macaronesia; U = Europe; A = Asia. The complete probabilities of each biogeographical region are presented in S3 Fig.

When considering a unique substitution rate (analysis I), dating analyses indicate that the split of *O*. *shevockii* between California and Tenerife populations dates back to the late Miocene–Pliocene (2.75 Ma; 95% highest posterior density interval (HPD): 0.44–6.69 Ma, Fig 8 node A). The results considering different rates for the nuclear and plastid partitions (analysis II) place the split 1.74 Ma (95% HPD: 0.17–3.73 Ma, S2 Fig node A). The common ancestor of the Californian clade dates back to 24.4 Ma (95% HPD: 10.9–31.45 Ma, Fig 8 node D) in analysis I and 18.44 Ma (95% HPD: 10.37–28.1 Ma, S2 Fig, node D) in analysis II. The best-fit model of ancestral area estimations, the DEC (Table 3), suggests that the present distribution of *O*. *shevockii* results from at least one long-distance dispersal event from western North America, which is supported by its inclusion in a highly supported western North American clade along with, as mentioned before, the Canary Island endemic *O*. *handiense* (Fig 7). When running the DEC analyses on 100 BEAST trees randomly sampled from the posterior probability distribution, the ancestral area estimates for the clades of interest (i.e. *O*. *shevockii*) were fully consistent with the former analysis (S3 Fig).

**Table 3:** Performance of competing models of ancestral-area estimation according to BioGeoBEARS.

	ln*L*	*n*	*d*	*e*	AIC
DEC	**-74.0978**	**2**	**0.0065**	**10**^**−12**^	**152.2**
DIVALIKE	-74.3172	2	0.0075	10^−12^	152.6
BAYAREALIKE	-80.9334	2	0.0045	0.02	165.9

The best model is highlighted in bold. Performance assessed by lnL (log-likelihood) and AIC (Akaike information criterion). DEC, dispersal–extinction–cladogenesis; DIVA, dispersal–vicariance analysis; *n*, number of parameters; *d*, rate of dispersal; *e*, rate of extinction.

## Discussion

### Taxonomic relationships of Orthotrichum shevockii and O. kellmanii

Our results show a lack of morphological or molecular differences that would have supported the consideration of *O*. *shevockii* and *O*. *kellmanii* as separate species, based on the analysis of a significant number of samples and including type materials from both taxa. The morphological analyses revealed that a large number of qualitative traits exhibited a uniform variation range among these specimens. These traits include some basic qualitative traits regarding the gametophyte and sporophyte, but also others that are singular within the genus *Orthotrichum* [33,44,46,78], and thus can be considered as diagnostic characters, such as: stomata restricted to the neck, exostome lacunose, and endostome PPL ornamented with lines or striae (Fig 4). Moreover, quantitative traits did not reveal any evidence of taxonomic differentiation (Fig 5), and molecular analyses resulted in grouping the different samples ascribable to these two Californian species in a well-supported clade. Within this group, further segregation of samples seems to have no geographical, ecological or taxonomical meaning.

Our findings do not support the previous consideration of *O*. *shevockii* and *O*. *kellmanii* as two different species, and probably points to the fact that the description of both taxa was made based on very few samples, which exhibited extremes of the morphological variation with respect to some gametophytic traits. *Orthotrichum shevockii* was described [39] based on samples from two close inland xeric localities at the junction of the southern Sierra Nevada and western Mojave Desert, whose specimens have leaves with bistratosity mostly restricted to the margins. *Orthotrichum kellmanii* was described [40] based upon samples from two nearby coastal localities in which specimens showed completely bistratose leaves. Additionally, gametophores of these latter samples were larger than usual. In fact, these coastal localities of *O*. *kellmanii* are at considerably lower altitudes and receive more humidity due to the Pacific Ocean influence, with occasional summer fog, which could favor the greater development and shoot size of these populations. Under these circumstances, specimens of this moss species form long sympodial gametophytic axes with: (i) abundant basal short and ligulate leaves (identified as vegetative by [40]); (ii) and upper leaves (from female axes) that are progressively larger and lanceolate. This could explain why [40] interpreted the habit as weakly cladocarpic with extreme heterophylly, as the plants spread prostrate across the smooth sandstone rock surface. The development of shorter and somewhat different basal leaves, although rarely highlighted, is a common characteristic in *Orthotrichum* and related genera (see for example [44]), whereas true heterophylly related to male and female branches has only been reported for one European moss [79]. Considering all of these arguments, it is clear that there is no morphological or molecular evidence that would further support the separation of *O*. *kellmanii* and *O*. *shevockii* as two distinct species. Therefore, we propose that *O*. *kellmanii* be synonymized under the priority name of *O*. *shevockii*.

*Orthotrichum shevockii* Lewinsky-Haapasaari & D.H. Norris, Bryologist 101(3): 435. 1998.

= *Orthotrichum kellmanii* D.H.Norris, Shevock & Goffinet, Bryologist 107(2): 210. 2004. **syn. nov.**

### Taxonomic status of Tenerife populations

Once the circumscription of *O*. *shevockii* has been clarified, including the synonymization of *O*. *kellmanii* therein, we can now consider the identity of the Tenerife populations. Our analyses reveal that the moss populations newly found in the Canary Islands (Tenerife island) also correspond to *O*. *shevockii*. Specimens from these populations are rather uniform in gametophyte and sporophyte characteristics, and fall within the range of morphological variation encountered in western North America. As mentioned above, the fact that all samples from the Canary Islands and western North America share some diagnostic qualitative traits considered unusual within the genus (see Fig 4), along with their strong phylogenetic relatedness, is highly suggestive that these lineages represent the same species, from an evolutionary point of view.

Indeed, the molecular results (Fig 7), although based on only four molecular markers, with three of them belonging to the chloroplast compartment, agree with the morphological evidence, with the Canarian clade being nested within the Californian one. Additionally, ecological aspects point in the same direction. In Tenerife, for instance, *O*. *shevockii* occurs in arid areas at high altitudes, as a saxicolous moss associated with crevices, rock ceilings, and vertical faces on volcanic rocks. This is also the most frequent (micro-) ecological setting of *O*. *shevockii* in western North America, except for the occurrence of all the inland populations on acidic granitic (granodiorite) rocks, while the few coastal mountain localities are on a wide variety of rock types, from metavolcanics to marble.

The discovery of *O*. *shevockii* as new to the Canary Islands raises the number of species of Orthotrichaceous mosses to 14 (4 *Lewinskya*, 9 *Orthotrichum*, 1 *Pulvigera*) known for this archipelago [80,81]. It is evident that the increasing implementation of integrative taxonomic approaches substantially improves our knowledge of the real regional diversity of plant groups with reduced morphologies like mosses, and, in general, of the still incomplete cryptogamic floras of oceanic biogeographic regions like Macaronesia [14].

### California–Macaronesia disjunction of *Orthotrichum shevockii*

Our results lead to the conclusion that the distribution of *O*. *shevockii* is disjunct and includes western North America (California and Nevada) and Macaronesia (Canary Islands, Tenerife). The connection of cryptogamic floras of Macaronesia and America has already been described, mainly referring to species present on the Azores and Madeira archipelagos, and involving disjunctions with tropical or Caribbean regions [14,82]. Some of these species have their main distribution in America, similar to *O*. *shevockii*. However, the disjunction reported here between the Californian region and the Canary Islands is quite uncommon. Other spore-producing organisms such as lichens, also have species with this type of distribution [83], but among bryophytes, species that are present in both regions tend to also expand their distribution into the Mediterranean basin [45,84,85].

The dating analyses place the colonization of Tenerife directly from western North America between 2.75 Ma (95% HPD: 0.44–6.69 Ma, analysis I) and 1.74 Ma (95% HPD: 0.17–3.73 Ma, analysis II), long after Tenerife island (11.9 Ma), and even the central and larger parts of the island (3 Ma) [86,87], where *O*. *shevockii* currently occurs, were formed. The phylogenetic inferences resolved *O*. *shevockii* within a clade composed of Californian endemic species, and the ancestral area estimation suggests a western North American origin for its ancestor (Figs 7 and 8). Our findings thus support that the present distribution of *O*. *shevockii* is the result of a long-distance dispersal event from California to the Canary Islands. This confirms the hypothesis that recurrent events of long-distance dispersal have occurred within the genus *Orthotrichum* from western North America (California) to the Macaronesian region, and in particular to the Canary Islands [25]. These events have taken place at different times and reflect different dispersal windows, with the split of *O*. *underwoodii* and *O*. *handiense* being older [25] than the disjunction of *O*. *shevockii* (Fig 8).

The Californian origin of *O*. *shevockii* provides additional support for the hypothesis that the Macaronesian cryptogamic flora may be more related to the New World [14], at least for certain groups of bryophytes, whereas angiosperms are more related to Europe and North Africa [13]. Moreover, it increases the evidence for bryophyte species with transoceanic distributions, which includes a number of Macaronesian taxa [25,82,88]. In the case of the Canary Islands, trade winds that cross the Atlantic Ocean run from east to west—opposite to the direction that has been identified for this long-distance dispersal—and cannot explain this disjunction. On the contrary, the high altitude subtropical jet stream that crosses over California and the Canary Islands [89,90] seems to be a suitable vector for wind-mediated dispersal events from west to east, as has been suggested in general for long-distance wind dispersal events in bryophytes [91] and, in particular for the North America-Europe disjunction [92].

Although *O*. *shevockii*’s range is constrained to few scattered locations in western North America (mountainous areas of California and nearby regions of westernmost Nevada), the presence of the species in the Canary Islands is restricted to a significantly smaller area. Restricted ranges in bryophytes are related to a recent origin, loss or lack of dispersal ability, extinction, preference for a specific habitat or a combination of some of these factors [92]. Our dating analyses do not rule out a relatively recent origin for the disjunction, which could be placed between 0.17 and 6.69 Ma (see above). Concerning dispersal capabilities, all collected samples showed sporophytes with high numbers of spores that are small enough (ca. 12 μm in diameter), a particular size thought to be easily carried by wind over long distances [10,93]. Furthermore, it has been suggested that Macaronesian bryophyte species do not necessarily lose their dispersal ability, maintaining connections between islands, archipelagos and nearby continents [24,94–97]. Therefore, its restricted area is not a priori attributed to reproductive constraints, but likely to habitat limitations.

Most of the bryophyte species (endemic or otherwise) with restricted distributions in the Canary Islands grow in very rare habitats, which is especially observed among taxa restricted to the laurel forest or inhabiting high altitude scrublands [82]. The latter match the distribution of *O*. *shevockii* in Tenerife, since it only occurs on rocks in open arid zones dominated by leguminous scrubs at altitudes around 2100 m a.s.l. On this archipelago, these altitudes are also found in La Palma Island, where the favorable habitat for this moss is more restricted than in Tenerife. This type of habitat is absent from the Azores and Cape Verde, the other Macaronesian archipelagos that exceed elevations of 2000 m a.s.l. [98]. Therefore, the distribution of *O*. *shevockii* in Macaronesia, which is currently restricted to Tenerife, could be explained by the lack of a suitable habitat in the region, a relatively recent founding event or a combination of these two phenomena. Future research on the landscape population genetics of disjunct lineages like *O*. *shevockii* represent a unique opportunity to improve our mechanistic understanding of origin, evolution and distribution of insular bryophyte floras.

## Supporting information

S1 Appendix Selection of samples used for morphological analyses. DNA ID numbers correspond to the specimens included in molecular analyses as used in Fig 1 and S2 Appendix.
(PDF)

S2 Appendix Specimens included in the molecular analyses and GenBank accession numbers. New accessions from this study are in italics. Numbers between brackets after taxon ID correspond to the specimens included in molecular analyses as used in Figs 1 and 7. Samples originally identified as *Orthotrichum kellmanii* appear under this name in the table.
(PDF)

S1 Fig Results of the principal component analysis (PCA) representing the first three components. The percentage of variance explained by each component is given in brackets. Arrows represent the variables included in the analyses. cos2 represents the squared loadings for variables. ! = samples originally identified as *Orthotrichum kellmanii*.
(TIF)

S2 Fig Molecular dating using a distinct nuclear and plastid nucleotide substitution rate. Maximum clade credibility tree from the relaxed molecular-clock analysis of the four loci in BEAST from analysis II with a distinct rate for the plastid (5.0E^-4^ (2–8E^-4^) subst./site/ma) and nuclear partitions (4.13E^-3^ (1.72–8.34E^-3^) subst./site/ma). Black and grey circles at nodes refer to node support of PP >0.95 and PP >0.75 - <0.95, respectively. Identification number and geographical origin follow sequence labels. In the case of *Orthotrichum shevockii*, sequence labels are also followed by number identification between brackets as in Fig 1 and S2 Appendix (*** = isotype material of *O*. *kellmanii*).
(TIF)

S3 Fig Biogeographical analyses. Ancestral area reconstructions. Chronogram of the phylogenetic relationships among the four loci from analysis I, and ancestral area estimations for the *Orthotrichum shevockii* group and the evaluated ingroup estimated from analyses run using 100 BEAST trees randomly sampled from the posterior probability distribution. Pie charts show the relative probability of each area or combination of areas being ancestral, according to the ancestral area reconstructions under the DEC model implemented in BioGeoBEARS. Ancestral states: global optim, 6 areas max.; d = 0.0065; e = 0; LnL = −75.09. Letters in coloured boxes correspond to the following ancestral areas or combination of areas: W = western North America; E = Eastern North America; N = Neotropics; M = Macaronesia; U = Europe; A = Asia.
(TIF)

S1 Table Quantitative morphological data. (XLSX)

S2 Table Marginal likelihood (MLE) and Bayes factor (BF) values for alternative clocks and models tested in BEAST. The best model is marked in bold.
(PDF)

S3 Table Pairwise nucleotide differences among the ingroup sequences of the *Orthotrichum shevockii* group. (PDF)
