# Supplementary material for: The long journey of Orthotrichum shevockii (Orthotrichaceae, Bryopsida): From California to Macaronesia
Source: PLoS One. 2019 Feb 13;14(2):e0211017. doi: 10.1371/journal.pone.0211017 (PMC6373912; doi:10.1371/journal.pone.0211017)
Supplement: S1 Appendix — DNA ID numbers correspond to the specimens included in molecular analyses as used in Fig 1 and S2 Appendix. (PDF) [file pone.0211017.s001.pdf]

Region/County	Locality	Collector	Collection date	Herbarium ID	DNA ID
**Spain: Canary Islands, Tenerife**					
P.N. del Teide	Cuadrícula 208	J.M.B., J.G.M. & J.L.P s.n.	27 Mar 2008	TFC-Bry 15957	
P.N. del Teide	O del Valle de Chiñoque y al N de Montaña de La Cruz	Losada-Lima s.n.	17 Oct 2008	TFC-Bry 16998	
P.N. del Teide	Barranco de La Zarza, base Montaña Los Asientos	Losada-Lima s.n.	18 Apr 2008	TFC-Bry 15633	
P.N. del Teide	Cañada del Montón de Trigo	Losada-Lima, León & Díaz s.n.	1 Jul 2008	TFC-Bry 15952	
P.N. del Teide	Ladera del Teide, bajo Piedras Negras	Losada-Lima s.n.	25 Apr 2008	TFC-Bry 15858	[15]
P.N. del Teide	Montaña Blanca, laderas del Teide	Losada-Lima, León & Díaz s.n.	2 Jul 2008	TFC-Bry 15904	[12]
P.N. del Teide	Montaña Blanca, laderas del Teide	Losada-Lima, León & Díaz s.n.	2 Jul 2008	TFC-Bry 15861	[13]
P.N. del Teide	Montaña Blanca, laderas del Teide	Losada-Lima, León & Díaz s.n.	2 Jul 2008	TFC-Bry 15909	[14]
P.N. del Teide	Montaña de los Pinos (de Arriba)	Losada-Lima s.n.	06 Mar 2009	TFC-Bry 17428	[16]
P.N. del Teide	Montaña de los Valles	Losada-Lima s.n.	06 Mar 2009	TFC-Bry 17406	
P.N. del Teide	Próximo a Diente del Risco	Losada-Lima s.n.	11 Apr 2008	TFC-Bry 15567	
**USA: California**					
Calaveras Co.	Cave City Road, north of Dirty Gulch	Norris 103406	20 Jan 2002	UC 1767898	
Kern Co.	Off of the Pacific Crest Trail, south of Walker Pass to fork of Jack Creek, Kiavah Wilderness, Scodie Mountains	Shevock 13404 & York 13404	11 May 1996	CAS 958716 **	[2]
Lake Co.	East of Round Mountain along Jerico Creek	Toren 7061	26 Apr 1998	MAUAM 3288	
Monterey Co.	Los Padres Nat. Forest	Shevock 29890	23 May 2007	CAS 1083201	
Monterey Co.	Los Padres Nat. Forest, Central Coast Ranges, Santa Lucia Range, Ventana Wilderness, south of Marble Peak	Shevock 32935, Kellman & Lodder	23 May 2009	MAUAM 5097	[9]
Monterey Co.	Los Padres Nat. Forest, Central Coast Ranges, Santa Lucia Range, Ventana Wilderness, south of Marble Peak	Shevock 32935, Kellman & Lodder	23 May 2009	NY 01140598	[10]
Mono Co.	Benton Range, west of Benton Hot Springs	Shevock 22289 & Glazer	28 May 2002	UC 1754201	
Mono Co.	Slinkard Creek, South of Topaz lake	Shevock 21802 & Glazer	20 Feb 2002	UC 1754431	[6]
Riverside Co.	San Bernardino Nat. Forest, San Jacinto Mts., Bay Tree Spring	Lara, Garilleti & Shevock s.n.	16 Nov 2008	MAUAM 3291	[1]
San Bernardino Co.	San Bernardino Nat. Forest, San Gorgonio Mts., Laurel Pines Camps	Lara, Mazimpaka & Vigalondo s.n.	1 Jan 2013	MAUAM 3313	
Santa Cruz Co.	Big Basin Redwoods State Park, near Basin Trail and China Grade	Kellman 1251	21 Jan 2001	CONN 00053520 ***	[11]
Shasta Co.	Cassel-Fall River road south of Fall River Hills	Norris 84716	3 Feb 1995	UC 1774443	
Shasta Co.	Cassel-Fall River road south of Fall River Hills	Norris 84724	4 Feb 1995	UC 1774462	[8]
Tulare Co.	Trail to Crystal Cave in the vicinity of Cascade Trail	Shevock 15768 & Tseng	15 Jun 1997	UC 1711731	[4]
Tulare Co.	Sequoia Nat. Forest, Greenhorn Mountains, west of McNallys Fairview	Shevock 16754 &Anderson	11 Dec 1997	UC 1754230	[3]
Ventura Co.	Along Howard Ck.	Norris 55512	30 Dec 1979	UC 1649705	
**USA: Nevada**					
Carson City Co.	Carson Range, Voltaire Canyon	Shevock 21948	5 Apr 2002	UC 1754323	
Carson City Co.	Carson Range, Voltaire Canyon	Shevock 21949	6 Apr 2002	CAS 1040048	
Carson City Co.	Lake Tahoe Basin, Forest Service Vista Point	Shevock 22038	20 Apr 2002	CAS 1040116	
Carson City Co.	Lake Tahoe Basin, Forest Service Vista Point	Shevock 22038	20 Apr 2002	UC 1754264	[7]
Mineral Co.	Toiyabe Nat. Forest, Anchorite Hills near Anchorite Pass	Lara, Garilleti, Shevock & Albertos s.n.	28 Oct 2008	MAUAM 3289	[5]
Mineral Co.	Toiyabe Nat. Forest, Anchorite Hills near Anchorite Pass	Lara, Garilleti, Shevock & Albertos s.n.	28 Oct 2008	MAUAM 3290	

** = paratype of *O. shevockii* , ***= isotype of *O. kellmanii*
