# Supplementary material for: The long journey of Orthotrichum shevockii (Orthotrichaceae, Bryopsida): From California to Macaronesia
Source: PLoS One. 2019 Feb 13;14(2):e0211017. doi: 10.1371/journal.pone.0211017 (PMC6373912; doi:10.1371/journal.pone.0211017)
Supplement: S2 Appendix — New accessions from this study are in italics. Numbers between brackets after taxon ID correspond to the specimens included in molecular analyses as used in Figs 1 and 7. Samples originally identified as Orthotrichum kellmanii appear under this name in the table. (PDF) [file pone.0211017.s002.pdf]

				Genbank accession number			
**Taxon**	**ID**	**Locality**	**Voucher**	**ITS2**	***rps*** **4**	***trn*** **L-F**	***atp*** **B-*****rbc*** **L**
Macrocoma lycopodioides	BV024	South Africa, Western Cape	MAUAM 2953	KT862258	KT862288	*MH275523*	*MH275428*
Nyholmiella obtusifolia	O118	Spain, Burgos	MAUAM 4343	--	JQ836797	JQ836986	JQ836695
N. obtusifolia	O119	France, Haute-Savoie	MAUAM 4342	--	JQ836798	JQ836987	JQ836696
Lewinskya acuminata	BV006	France, Corse	MAUAM 3164	KT862262	KT862292	KT862321	*MH275429*
L. acuminata	BV010	Spain, Ávila	MAUAM 3272	KT862263	KT862293	KT862322	*MH275430*
L.affinis	BV015	Spain, Jaén	MAUAM 4448	KT862277	KT862306	KT862335	*MH275431*
L.fastigiata	BV016	Turkey, Artvin	MAUAM 4449	KT862278	KT862307	KT862336	*MH275432*
Orthotrichum alpestre	R669	Turkey, Gümüshane	MAUAM 4391	*MH275451*	JQ836864	JQ837053	JQ836760
O. alpestre	R670	Switzerland, St. Gallen	MAUAM 1685	*MH275452*	JQ836865	JQ837151	JQ836761
O. anomalum	O120	Spain, Asturias	MAUAM 4330	*MH275453*	JQ836799	JQ836988	JQ836697
O. bartrami	R598	USA, Arizona	CAS sn	*MH275454*	JQ836838	JQ837027	JQ836734
O. bistratosum	BV088	Spain, Jaén	MAUAM 4594	*MH275455*	*MH275504*	*MH275524*	*MH275433*
O. casasianum	R398	Spain, Álava	MAUAM 1702	*MH275456*	JQ836811	JQ837000	JQ836707
O. columbicum	R674	Canada, British Columbia	MAUAM 4284	*MH275457*	JQ836874	JQ837063	JQ836770
O. columbicum	R678	Spain, León	MAUAM 657	*MH275458*	JQ836877	JQ837066	JQ836773
O. comosum	R655	Spain, Almería	MAUAM 4359	*MH275459*	JQ836852	JQ837041	JQ836748
O. comosum	R673	Spain, Cadiz	MAUAM 4361	*MH275460*	JQ836860	JQ837049	JQ836756
O. confusum	R680	USA, California	MAUAM 4323	*MH275463*	JQ836878	JQ837067	JQ836774
O. consimile	R677	USA, California	MAUAM 4278	*MH275461*	JQ836869	JQ837058	JQ836765
O. consimile	R616	USA, California	UC 1760062	*MH275462*	JQ836870	JQ837059	JQ836766
O. coulteri	R564	USA, California	MAUAM 4367	*MH275464*	JQ836817	JQ837006	JQ836713
O. coulteri	R562	USA, California	MAUAM 4368	*MH275465*	JQ836815	JQ837004	JQ836711
O. cuculatum	R577	USA, California	MAUAM 4381	*MH275466*	JQ836830	JQ837019	JQ836726
O. cuculatum	R579	USA, California	MAUAM 4380	*MH275467*	JQ836829	JQ837018	JQ836725
O. flowersii	R632	USA, Nevada	CAS-1045756	--	JQ836842	JQ837031	JQ836738
O. franciscanum	R570	USA, California	MAUAM 4390	*MH275468*	JQ836823	JQ837012	JQ836719
O. franciscanum	R571	USA, California	UC 1739290	*MH275469*	JQ836824	JQ837013	JQ836720
O. hallii	BV089	USA, Nevada	MAUAM 4595	*MH275470*	*MH275505*	*MH275525*	*MH275434*
O. hallii	BV090	USA, Nevada	MAUAM 4596	*MH275471*	*MH275506*	*MH275526*	*MH275435*
O. handiense	O2115	Spain, Canary Is., Fuerteventura	MAUAM 4689	*MH275472*	JX297214	JX297224	JX297209
O. handiense	O2172	Spain, Canary Is., Fuerteventura	MAUAM 4690	*MH275473*	JX297215	JX297225	JX297210
O. handiense	O2173	Spain, Canary Is., Fuerteventura	MAUAM 2043	*MH275474*	JX297216	JX297226	JX297211
O. kellmanii = O.shevockii	BV054 [9]	USA, California	MAUAM 5097	*MH275475*	*MH275507*	*MH275527*	*MH275436*
O. kellmanii = O.shevockii	BV103 [10]	USA, California	NY 01140598	*MH275476*	*MH275508*	*MH275528*	*MH275437*
O. kellmanii = O.shevockii	1502 [11]	USA, California	CONN00053520	--	*MH275509*	*MH275529*	*MH275438*
O. norrisii	R567	USA, California	MAUAM 4395	*MH275477*	JQ836820	JQ837009	JQ836716
O. norrisii	R568	USA, California	UC 1741966	*MH275478*	JQ836821	JQ837010	JQ836717

Taxon	ID	Locality	Voucher	Genbank accession number			
				ITS2	*rps* 4	*trn* L-F	*atp* B-*rbc* L
O. persimile	R580	USA, California	UC 1650645	*MH275479*	JQ836833	JQ837022	JQ836729
O. persimile	R666	USA, California	MAUAM 4327	*MH275480*	JQ836857	JQ837046	JQ836753
O. pilosisimum	R640	USA, Nevada	MAUAM 4334	*MH275481*	JQ836845	JQ837034	JQ836741
O. pilosisimum	R644	USA, Nevada	MAUAM 4333	*MH275482*	JQ836847	JQ837036	JQ836743
O. pulchellum	R682	Canada, British Columbia	MAUAM 4336	*MH275483*	JQ836880	JQ837069	JQ836776
O. pulchellum	R684	Spain, Álava	MAUAM 4338	*MH275484*	JQ836882	JQ837071	JQ836778
O. pumilum	R152	Estonia	TU 170	--	JQ836802	JQ836991	JQ836700
O. scanicum	R018	Greece, Sterea Hellada	MAUAM 2166	*MH275485*	JQ836800	JQ836989	JQ836698
O. schimperi	R364	Tunisia, Aïn-Draham	MAUAM 2448	*MH275486*	JQ836810	JQ836999	JQ836706
O. schimperi	R656	USA, California	MAUAM 4339	*MH275487*	JQ836853	JQ837042	JQ836749
O. sharpii	R769	Mexico, Veracruz	MAUAM 4340	--	JQ837050	JQ836861	JQ836757
O. shevockii	BV030 [5]	USA, Nevada	MAUAM 3289	*MH275488*	*MH275510*	*MH275530*	*MH275439*
O. shevockii	BV031 [2]	USA, California	CAS 958716	*MH275489*	*MH275511*	*MH275531*	*MH275440*
O. shevockii	BV032 [8]	USA, California	UC 1774462	*MH275490*	*MH275512*	*MH275532*	--
O. shevockii	BV043 [4]	USA, California	UC 1711731	*MH275491*	*MH275513*	*MH275533*	*MH275441*
O. shevockii	BV044 [6]	USA, California	UC 1754431	*MH275492*	*MH275514*	*MH275534*	*MH275442*
O. shevockii	BV045 [3]	USA, California	UC 1754230	*MH275493*	*MH275515*	*MH275535*	--
O. shevockii	BV046 [7]	USA, Nevada	UC 1754264	*MH275494*	*MH275516*	*MH275536*	*MH275443*
O. shevockii	BV048 [1]	USA, California	MAUAM 3291	*MH275495*	*MH275517*	--	*MH275444*
O. shevockii	BV049 [12]	Spain, Canary Islands, Tenerife	TFCBry 15904	*MH275496*	*MH275518*	*MH275537*	*MH275445*
O. shevockii	BV050 [13]	Spain, Canary Islands, Tenerife	TFCBry 15861	*MH275497*	*MH275519*	*MH275538*	*MH275446*
O. shevockii	BV051 [16]	Spain, Canary Islands, Tenerife	TFCBry 17428	*MH275498*	*MH275520*	--	*MH275447*
O. shevockii	BV052 [14]	Spain, Canary Islands, Tenerife	TFCBry 15909	*MH275499*	*MH275521*	*MH275539*	--
O. shevockii	BV053 [15]	Spain, Canary Islands, Tenerife	TFCBry 15858	*MH275500*	*MH275522*	*MH275540*	*MH275448*
O. tenellum	R294	Italy, Sicily	MAUAM 4346	*MH275501*	JQ836805	JQ836994	JQ836703
O. tenellum	R295	Portugal, Tras os Montes e Alto Douro	MAUAM 4347	*MH275502*	JQ836806	JQ836995	JQ836704
O. underwoodii	R583	USA, California	MAUAM 4341	*MH275503*	JQ836835	JQ837024	JQ836731
Zigodon pentastichus	ID207	Argentina, Córdoba	MAUAM 2981	KT862260	KT862290	KT862318	*MH275449*
Z. viridissimus	ID208	United Kingdom	MAUAM 2910	KT862259	KT862289	KT862319	*MH275450*
