# Supplementary figures and images for: The long journey of Orthotrichum shevockii (Orthotrichaceae, Bryopsida): From California to Macaronesia
Source: PLoS One. 2019 Feb 13;14(2):e0211017. doi: 10.1371/journal.pone.0211017 (PMC6373912; doi:10.1371/journal.pone.0211017)

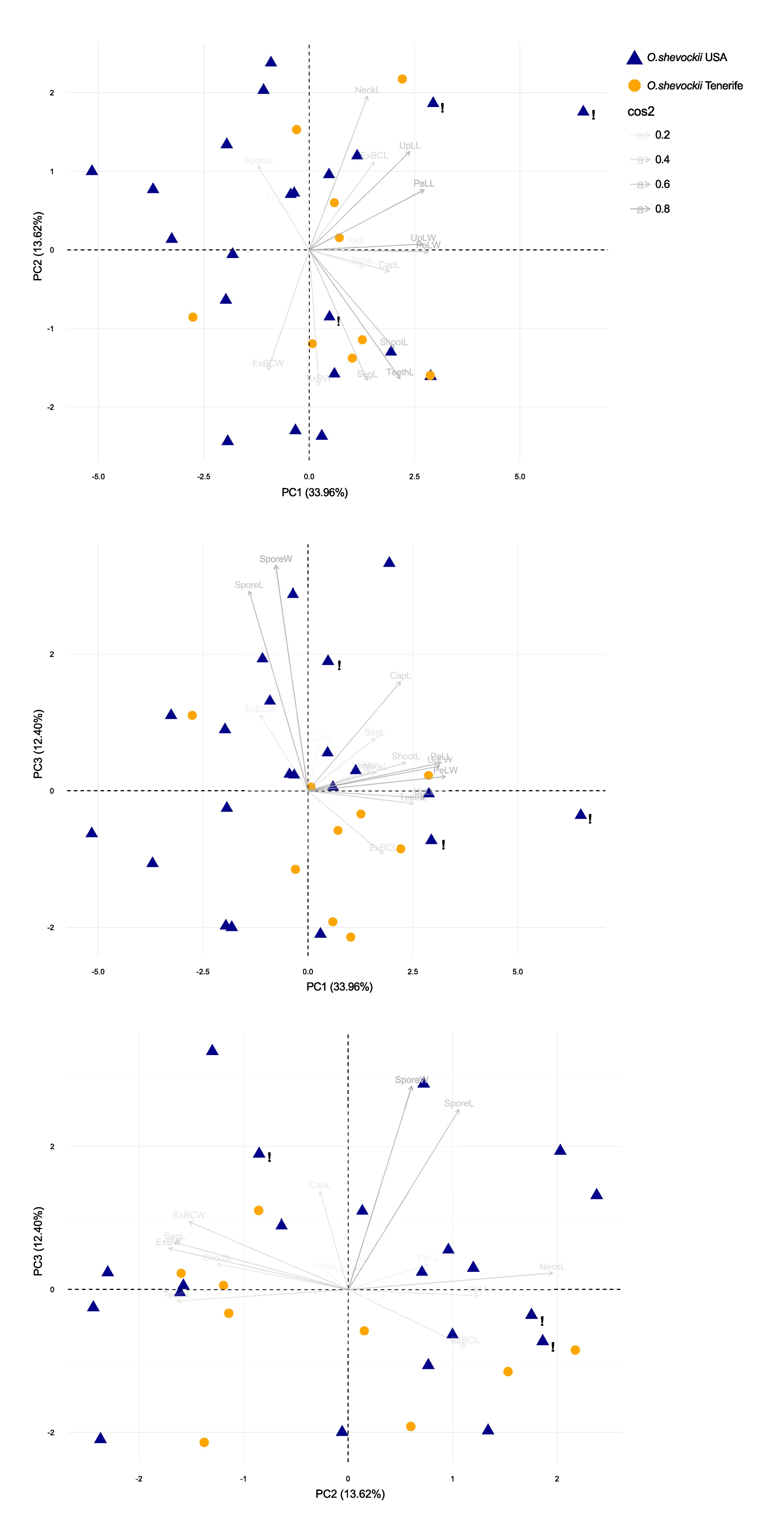

Supplement: S1 Fig — The percentage of variance explained by each component is given in brackets. Arrows represent the variables included in the analyses. cos2 represents the squared loadings for variables. ! = samples originally identified as Orthotrichum kellmanii. (TIF) [file pone.0211017.s003.tif]

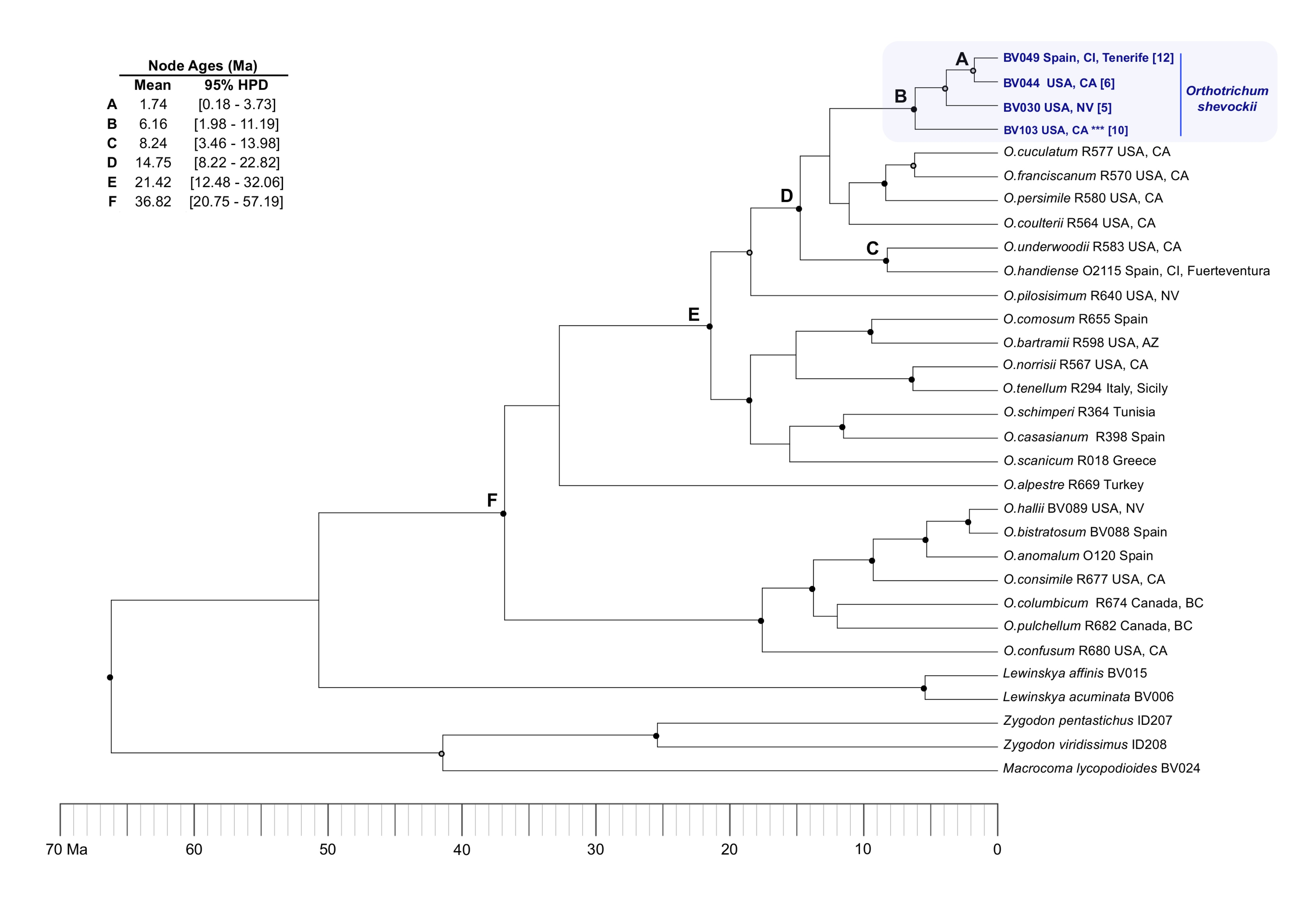

Supplement: S2 Fig — Maximum clade credibility tree from the relaxed molecular-clock analysis of the four loci in BEAST from analysis II with a distinct rate for the plastid (5.0E-4 (2–8E-4) subst./site/ma) and nuclear partitions (4.13E-3 (1.72–8.34E-3) subst./site/ma). Black and grey circles at nodes refer to node support of PP >0.95 and PP >0.75 - <0.95, respectively. Identification number and geographical origin follow sequence labels. In the case of Orthotrichum shevockii, sequence labels are also followed by number identification between brackets as in Fig 1 and S2 Appendix (*** = isotype material of O. kellmanii). (TIF) [file pone.0211017.s004.tif]

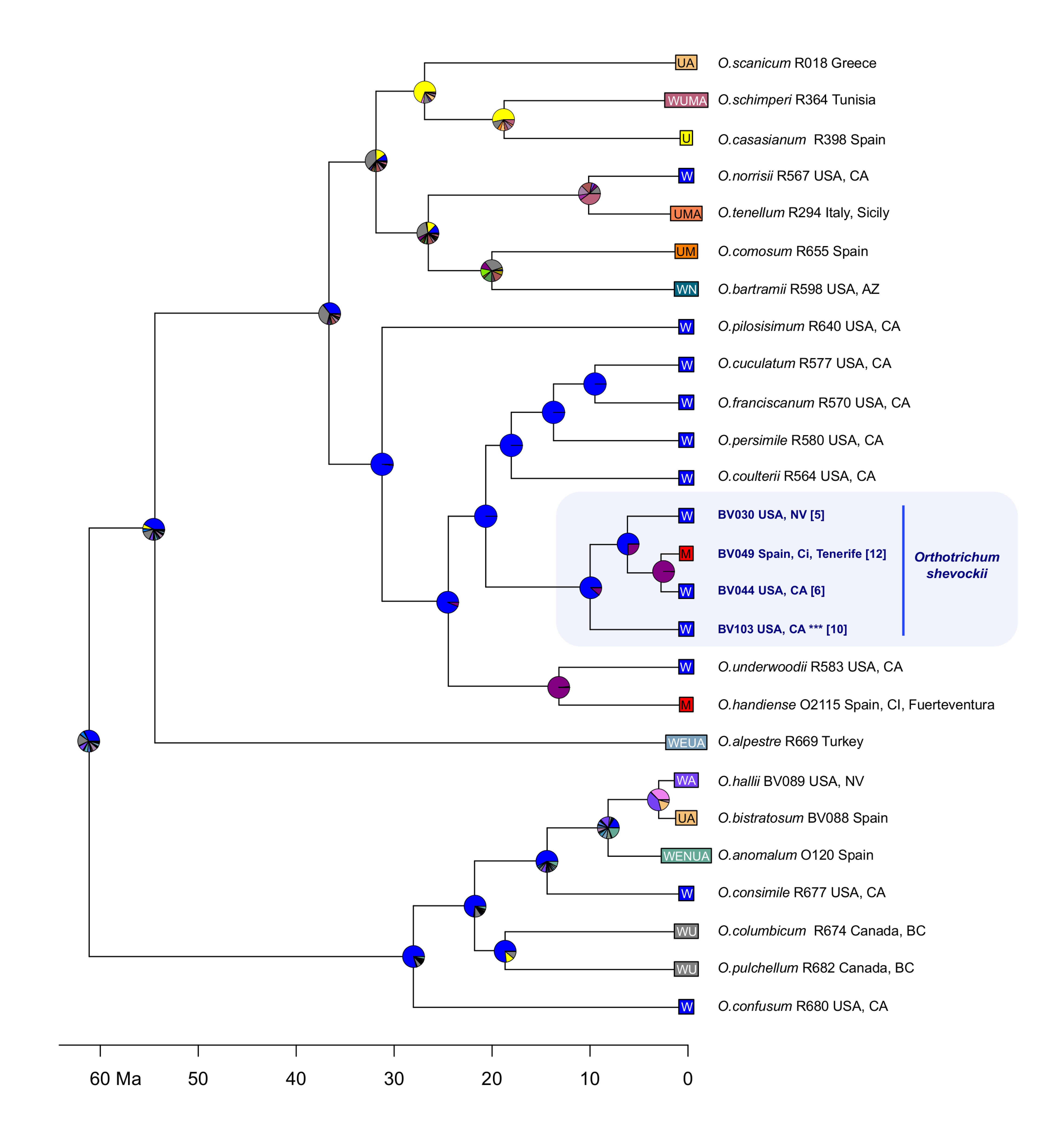

Supplement: S3 Fig — Ancestral area reconstructions. Chronogram of the phylogenetic relationships among the four loci from analysis I, and ancestral area estimations for the Orthotrichum shevockii group and the evaluated ingroup estimated from analyses run using 100 BEAST trees randomly sampled from the posterior probability distribution. Pie charts show the relative probability of each area or combination of areas being ancestral, according to the ancestral area reconstructions under the DEC model implemented in BioGeoBEARS. Ancestral states: global optim, 6 areas max.; d = 0.0065; e = 0; LnL = −75.09. Letters in coloured boxes correspond to the following ancestral areas or combination of areas: W = western North America; E = Eastern North America; N = Neotropics; M = Macaronesia; U = Europe; A = Asia. (TIF) [file pone.0211017.s005.tif]
