# Supplementary material for: The long journey of Orthotrichum shevockii (Orthotrichaceae, Bryopsida): From California to Macaronesia
Source: PLoS One. 2019 Feb 13;14(2):e0211017. doi: 10.1371/journal.pone.0211017 (PMC6373912; doi:10.1371/journal.pone.0211017)
Supplement: S2 Table — The best model is marked in bold. (PDF) [file pone.0211017.s007.pdf]

		Path Sampling		Stepping-Stone	
		ln (MLE)	2ln (BF)	ln (MLE)	2ln (BF)
Uncorrelated log-normal	**Birth-death**	**-5781,254**	**0**	**-5774,789**	**0**
	Yule	-5799,834	37,161	-5801,716	40,924
Strict consensus	Birth-death	-5819,606	76,704	-5820,374	78,239
	Yule	-5826,93	91,352	-5828,69	94,871
