# Supplementary material for: The long journey of Orthotrichum shevockii (Orthotrichaceae, Bryopsida): From California to Macaronesia
Source: PLoS One. 2019 Feb 13;14(2):e0211017. doi: 10.1371/journal.pone.0211017 (PMC6373912; doi:10.1371/journal.pone.0211017)
Supplement: S3 Table — (PDF) [file pone.0211017.s008.pdf]

	BV054 USA	BV103 USA	BV030 USA	BV031 USA	BV043 USA	BV044 USA	BV046 USA	BV049 TNF	BV050 TNF	BV053 TNF
BV054 USA	0									
BV103 USA	4	0								
BV030 USA	4	5	0							
BV031 USA	6	5	3	0						
BV043 USA	1	5	5	7	0					
BV044 USA	9	5	6	1	10	0				
BV046 USA	9	6	5	0	10	1	0			
BV049 TNF	7	7	3	2	9	4	3	0		
BV050 TNF	7	6	3	1	8	3	2	1	0	
BV053 TNF	7	7	3	2	9	4	3	0	1	0
